# Phosphorylation-Dependent Differential Regulation of Plant Growth, Cell Death, and Innate Immunity by the Regulatory Receptor-Like Kinase BAK1

**DOI:** 10.1371/journal.pgen.1002046

**Published:** 2011-04-28

**Authors:** Benjamin Schwessinger, Milena Roux, Yasuhiro Kadota, Vardis Ntoukakis, Jan Sklenar, Alexandra Jones, Cyril Zipfel

**Affiliations:** The Sainsbury Laboratory, Norwich Research Park, Norwich, United Kingdom; University of Toronto, Canada

## Abstract

Plants rely heavily on receptor-like kinases (RLKs) for perception and integration of external and internal stimuli. The Arabidopsis regulatory leucine-rich repeat RLK (LRR-RLK) BAK1 is involved in steroid hormone responses, innate immunity, and cell death control. Here, we describe the differential regulation of three different BAK1-dependent signaling pathways by a novel allele of BAK1, *bak1-5*. Innate immune signaling mediated by the BAK1-dependent RKs FLS2 and EFR is severely compromised in *bak1-5* mutant plants. However, *bak1-5* mutants are not impaired in BR signaling or cell death control. We also show that, in contrast to the RD kinase BRI1, the non-RD kinases FLS2 and EFR have very low kinase activity, and we show that neither was able to trans-phosphorylate BAK1 *in vitro*. Furthermore, kinase activity for all partners is completely dispensable for the ligand-induced heteromerization of FLS2 or EFR with BAK1 *in planta*, revealing another pathway specific mechanistic difference. The specific suppression of FLS2- and EFR-dependent signaling in *bak1-5* is not due to a differential interaction of BAK1-5 with the respective ligand-binding RK but requires BAK1-5 kinase activity. Overall our results demonstrate a phosphorylation-dependent differential control of plant growth, innate immunity, and cell death by the regulatory RLK BAK1, which may reveal key differences in the molecular mechanisms underlying the regulation of ligand-binding RD and non-RD RKs.

## Author Summary

Plants need to adapt to their ever-changing environment for survival. Transmembrane receptor kinases are essential to translate extracellular stimuli into intracellular responses. A key question is how plants maintain signaling specificity in response to multiple stresses and endogenous hormones. Growth responses induced by steroid hormones and innate immunity triggered by recognition of conserved microbial molecules depend on the common regulatory receptor-like kinase BAK1, which is also involved in cell death control. It is still unclear if BAK1 provides signaling specificity or if it is a mere signaling enhancer. Here, we describe the novel protein variant BAK1-5 that specifically blocks innate immune responses without affecting steroid responses or cell death. This unambiguously demonstrates that the role of BAK1 in plant signaling can be mechanistically separated. Importantly, the impairment of immune signaling is not caused by a loss of interaction of BAK1-5 with immune receptors but is due to an altered kinase activity. Thus, BAK1-dependent signaling pathways are under a differential phosphorylation-dependent regulation. The examination of this novel mutant version of BAK1 will enable detailed studies into the mechanistic role of BAK1 in plant innate immunity, but also more generally will provide invaluable insights into transmembrane receptor signaling specificity in plants.

## Introduction

Plants are under constant pressure to respond rapidly and accurately to changing environmental and developmental conditions. Hence they need to translate extracellular signals into appropriate intracellular responses. Cell surface receptor-like-kinases (RLKs) are one of the major components in this extracellular sensing. The model plant species Arabidopsis and rice show a huge expansion of the RLK family compared to other eukaryotes with >600 and >1100 members, respectively [1]. However, only a very limited number of plant RLKs have an assigned function ranging from development to responses to biotic and abiotic stresses [2]–[4].

Plant RLKs share a common domain organization with the well-studied mammalian receptor tyrosine kinases (RTKs) [5], [6]. The activation of RTKs is initiated by ligand binding to the extra-cellular domain leading to conformational changes that are transmitted by a single trans-membrane domain and induce receptor homo- and/or hetero-oligomerization [7]. This leads to activation by trans- and auto-phoshorylation of the activation loop, correct positioning of the cytoplasmic asymmetric kinase dimer, and release of the inhibition by the C-terminal and/or juxta-membrane regions [8]–[10]. Downstream signaling is initiated by sequential auto- or trans-phosphorylation of specific residues in the cytoplasmic domain serving as docking sites for downstream signaling partners, and/or direct phosphorylation of signaling substrates [11].

Kinases, including RLKs, can be subdivided into RD and non-RD kinases depending on the conservation of the amino-acid residue preceding the core catalytic aspartate (Asp) residue in subdomain VIb of the kinase domain [12], [13]. Most RD kinases require auto-phosphorylation of the activation loop for full kinase activity. In contrast, non-RD kinases do not require activation loop phosphorylation and are activated by different mechanisms [13].

Notably, several plant RD- and non-RD ligand-binding receptor kinases (RKs) share the common RD-type regulatory RLK BAK1 as signaling partner [14], [15]. The leucine-rich repeat (LRR)-RLK BAK1 (At4g33430) is a member of the somatic embryogenesis-related kinase (SERK) family and is also named SERK3 [16], [17]. BAK1 was initially identified as a positive regulator of brassinosteroid (BR) responses, forming a ligand-dependent complex *in vivo* with the LRR-RK BRI1 (At4g39400), the main BR receptor [18]–[21]. Over-expression of BAK1 suppresses weak *bri1* alleles, and *bak1* knock-out mutants are hypo-sensitive to BR and resemble weak *bri1* alleles [18], [19], [21].

BAK1 is also involved in cell death control as *bak1* knock-out mutants have a spreading lesion phenotype upon pathogen infection and premature senescence [22], [23]. This loss of cell death control is aggravated in double-mutant combinations with its closest paralog BKK1/SERK4 (At2g13790), and strong *bak1 bkk1* allele combinations are seedling lethal even in sterile conditions [22], [24]. Additionally, BAK1 interacts with BIR1 (At5g48380), another LRR-RLK, mutants of which also show constitutive uncontrolled cell death [25].

BAK1 was also identified as an important regulator of pathogen-associated-molecular-pattern (PAMP)-triggered immunity (PTI) [26], [27]. *Bak1* null mutants are compromised in their responsiveness to several PAMPs including flg22 (derived from bacterial flagellin), elf18 (derived from bacterial EF-Tu), HrpZ, lipopolysaccharides, peptidoglycans, and damage-associated molecular patterns (DAMPs), such as AtPep1 [26]–[29]. Furthermore, *BAK1*-silenced *Nicotiana benthamiana* (*N. benthamiana*) plants are less sensitive to the PAMPs INF1 and csp22 (derived from bacterial cold shock protein) [26]. BAK1 rapidly forms ligand-dependent heteromers with the flg22 and elf18 pattern-recognition receptors (PRRs), the ligand-binding LRR-RKs FLS2 (At5g46330) and EFR (At5g20480), respectively [26], [27], [30](Roux et al., *submitted*). BAK1 also interacts in a ligand-independent manner with the AtPep1 PRRs, the ligand-binding LRR-RKs AtPEPR1/2 (At1g73080/At1g17750) in yeast two-hybrid assays [15]. The importance of the heteromerization with BAK1 in plant innate immunity is substantiated by the targeting of the ligand-induced BAK1-FLS2 interaction by the bacterial virulence effector AvrPto to block PTI signaling [29], [31], [32] Importantly, the function of BAK1 in cell death control and innate immunity seems to be independent of its function in BR signaling [14].

Clearly, BAK1 is an important regulator implicated in multiple independent signaling pathways leading to growth, cell death control and innate immunity. Although BAK1 forms ligand-dependent heteromers with several ligand-binding LRR-RKs [20], [21], [26], [27], it is not required for ligand binding [27], [33]. In that respect, BAK1 should be considered as a regulatory RLK rather than a co-receptor. It is, however, not fully understood how BAK1 regulates these different pathways.

A previous study suggests that BAK1 functions as a signal enhancer for the RD-kinase BRI1 [21]. This conclusion is based on biochemical studies into auto- and trans-phosphorylation events revolving around BRI1-BAK1 followed by phenotypic analysis of BAK1 phospho-mimetic and phospho-dead mutants. Interestingly none of the BAK1 mutant alleles had a strong differential effect on PTI and BR signaling [21]. The activation of BAK1 by BRI1 is further supported by a recent report showing that a tyrosine auto-phosphorylation site in the C-terminus of BAK1 is required for trans-activation of BRI1 [34]. Interestingly, this auto-phoshorylation site of BAK1 is not required for flg22-induced seedling growth inhibition (SGI) [34]. Given this differential requirement of phosphosites and the different mode of regulation of non-RD kinases versus RD kinases [12], [13], it is unclear whether the BRI1-BAK1 model can be generalized to non-RD kinases. Since non-RD kinases are mostly associated with functions in innate immunity across kingdoms [35], it is of great interest to elucidate potential regulatory mechanisms of non-RD kinases and to reveal potential differences to RD kinases.

Here, we demonstrate the phosphorylation-dependent differential regulation of the RD-kinase BRI1 and the non-RD kinases FLS2 and EFR by BAK1. We identified a novel mutant allele of *BAK1*, *bak1-5*, that is strongly impaired in PTI signaling but displays a wild-type-like BR signaling capacity. Furthermore, *bak1-5* is not impaired in cell death control. This unexpected phenotype is not due to a differential complex formation between BAK1-5 and the RD and non-RD kinases, but requires the kinase activity of BAK1-5 suggesting a phosphorylation-dependent differential regulation. Moreover, our work reveals dramatic differences in the trans-phosphorylation events between BAK1 and BRI1 or EFR *in vitro*, and the requirement of kinase activity for complex formation *in planta*.

## Results

### Identification of the novel *BAK1* allele *bak1-5*

**Figure 1:**
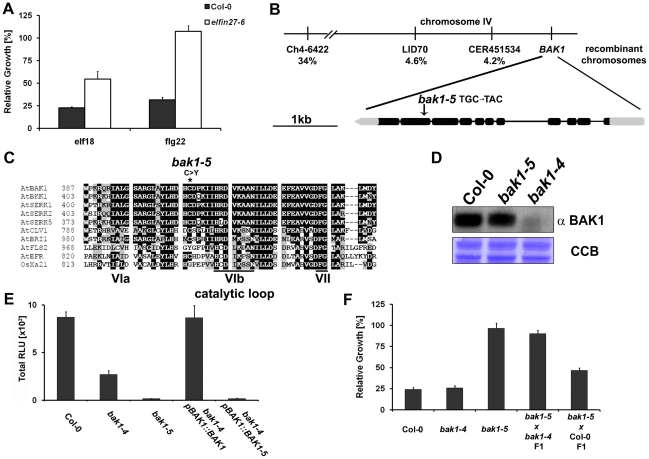
*elfin27-6* is a novel allele of *BAK1.* A. *elfin27-6* is impaired in seedling growth inhibition triggered by 60 nM elf18 or flg22. Fresh weight is represented relative to untreated control. Results are average ± s.e (n = 6). B. *elfin27-6* carries a single mis-sense mutation in the 10^th^ exon of *BAK1*. Schematic representation of relative marker positions and observed recombination rates in a L*er*-0×*elfin27-6* F2 mapping population. C. Cys408 is conserved in all AtSERK family members but not in all RLKs. Alignment of kinase subdomains VIa, VIb and VII of AtSERKs, AtBRI1, AtCLV1, AtFLS2, AtEFR and OsXA21. The star indicates the Cys to Tyr change in BAK1-5. D. BAK1-5 accumulates to wild-type levels. Immunoblot of total protein from Col-0, *bak1-5* and *bak1-4* using anti-BAK1 antibody. Immunoblot, upper panel; Coomassie colloidal blue stained membrane, lower panel. E. The *bak1-5* mutation is causative for the reduced flg22-induced ROS burst. Total ROS burst in leaves of Col-0, *bak1-4*, *bak1-5*, *bak1-4 pBAK1::BAK1*, and *bak1-4 pBAK1::BAK1-5* after treatment with 100 nM flg22. Results are average ± s.e. (n = 8). F. *bak1-5* is a semi-dominant allele. Seedling growth inhibition of Col-0, *bak1-4*, *bak1-5*, *bak1-5×bak1-4* F1 and *bak1-5*×Col-0 F1 in the presence of 10 nM elf18. Fresh weight is represented relative to untreated control. Results are average ± s.e (n = 6). These experiments were repeated at least three times with similar results.

To identify novel regulators of EFR function/signaling in *Arabidopsis thaliana*, we previously performed a forward-genetic screen for *elf18-insensitive* (*elfin*) mutants based on loss SGI triggered by elf18 [36]. Out of 103 non-*efr elfin* mutants recovered, only one, *elfin27-6*, showed a clear defect in the SGI induced by both elf18 and flg22, even at high peptide concentrations (1 mM) (Figure 1A, Figure S1). This suggested that this mutant was affected in an important component shared by both EFR- and FLS2-dependent signaling pathways.

Using a map-based cloning approach we identified the corresponding mutation as a single mis-sense substitution in the 10^th^ exon of *BAK1* (Figure 1B). We therefore tentatively renamed *elfin27-6* as *bak1-5*. This mutation leads to a C408Y change in the subdomain VIa of the cytoplasmic kinase preceding the catalytic loop (Figure 1C). This Cys residue is conserved in ∼17% of all RLKs in *Arabidopsis thaliana* (data not shown).

Next, we tested whether the *bak1-5* mutation affects the accumulation of the BAK1 protein. To this end, we performed immunoblot analysis on protein extracts of Col-0, *bak1-5* and *bak1-4* (SALK_116202) mutant plants using anti-BAK1 antibodies. As shown in Figure 1D, full-length mutant BAK1-5 protein accumulated to similar levels as the wild-type protein, whereas the corresponding band was completely missing in *bak1-4* null mutants.

To confirm that the C408Y mutation causes the observed *elfin* phenotype, we first transformed the null mutant *bak1-4* with *BAK1* or *BAK1-5* genomic sequences under the control of their own regulatory sequences. As expected, the wild-type transgene was able to complement the compromised flg22- and elf18-induced reactive oxygen species (ROS) burst of *bak1-4* (Figure 1E and Figure S2A–S2B). Consistently, transgenic plants expressing *BAK1-5* were strongly impaired in flg22- and elf18-induced ROS burst and thus phenocopied the *bak1-5* mutant (Figure 1E and Figure S2A–S2B).

To further prove that the *bak1-5* mutation causes the *elfin* phenotype, and to ascertain whether *bak1-5* is a recessive or dominant mutation, we took advantage of the fact that *bak1-4*, in contrast to *bak1-5* (Figure 1A and Figure S1), is not impaired in the SGI triggered by elf18 (Figure 1F and Figure S1) [27]. We tested the contribution of BAK1-5 to the impaired elf18-induced SGI in an allelism test between *bak1-5* and *bak1-4*. Only homozygous *bak1-5* and *bak1-5*×*bak1-4* heterozygous F1 seedlings showed a strong impairment in elf18-induced SGI (Figure 1F). Interestingly *bak1-5*×Col-0 heterozygous F1 plants showed an intermediate phenotype between wild-type Col-0 and *bak1-5* seedlings (Figure 1F). This indicates that *bak1-5* is a semi-dominant allele and suggests that BAK1-5 has as a dose-sensitive dominant-negative effect on the endogenous wild-type BAK1. This semi-dominant-negative effect was not restricted to SGI, but was also observed when elf18-induced ROS burst was measured in leaves of *bak1-5*×Col-0 heterozygous F1 plants (Figure S2C).

Therefore, *bak1-5* is a novel semi-dominant allele of *BAK1* with a specific phenotype related to PAMP responsiveness.

### *bak1-5* is strongly impaired in EFR- and FLS2-dependent PTI signaling

Previous results showed that the null *bak1-4* mutant plants were strongly impaired in early and late responses to flg22, but were not impaired in late elf18 responses [27]. In particular, elf18-induced SGI in *bak1-4* was indistinguishable from wild-type (Figure 1 E and Figure S1) [27]. Since the novel allele *bak1-5* was impaired in both flg22- and elf18-triggered SGI, we investigated the impact of the *bak1-5* mutation on early and late responses triggered by flg22 and elf18.

**Figure 2:**
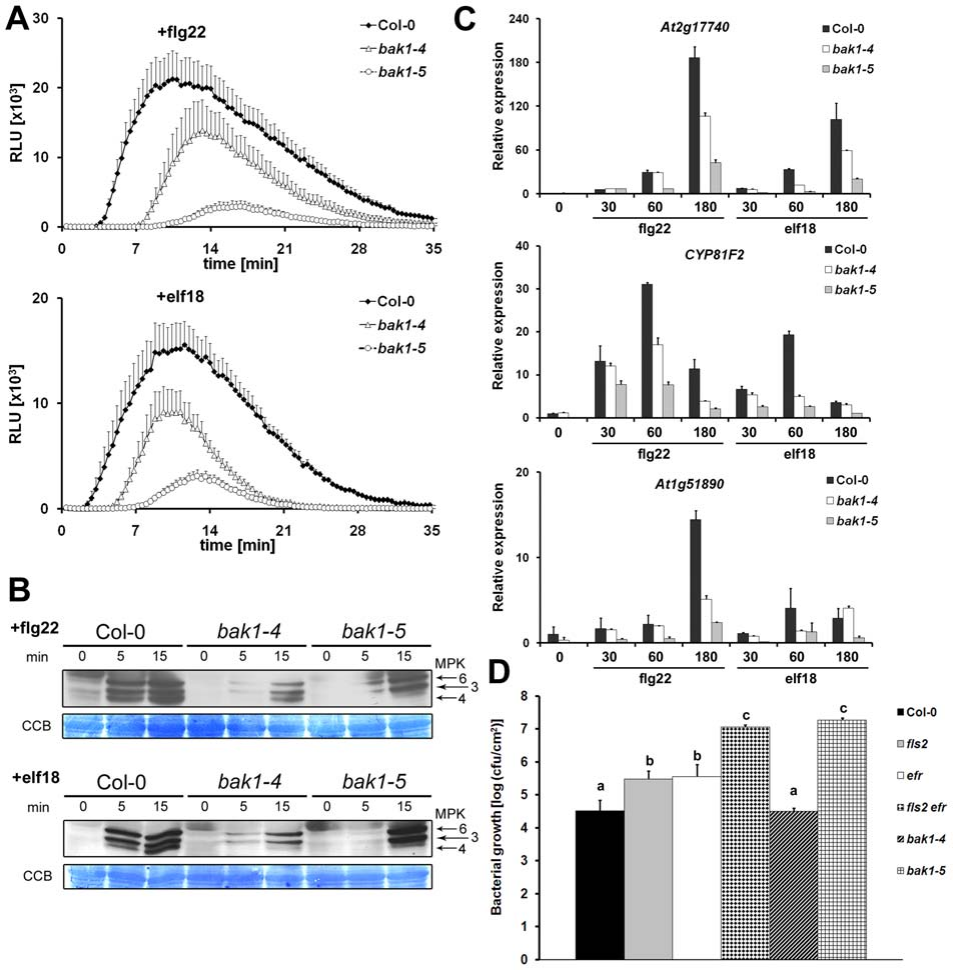
*bak1-5* is strongly impaired in EFR- and FLS2-dependent PTI signaling. A. *bak1-5* is strongly impaired in flg22- and elf18-induced ROS burst. ROS burst in leaves of Col-0, *bak1-4*, *bak1-5* after treatment with 100 nM flg22 (upper panel) or elf18 (lower panel). Results are average ± s.e. (n = 8). B. Differential MPK activation in *bak1-5* after flg22 and elf18 treatment. The kinetics of kinase activation in seedlings of Col-0, *bak1-4* and *bak1-5* treated with either 100 nM flg22 (upper panel) or elf18 (lower panel) as shown by immunoblot analysis using an anti-p44/42-ERK antibody. Individual MPKs are identified by molecular mass and indicated by arrows. Immunoblot, upper panel; Coomassie colloidal blue stained membrane, lower panel. C. Defence gene induction by flg22 and elf18 is strongly impaired in *bak1-5*. Gene expression of *At2g17740* (upper panel), *CYP81F2* (middle panel) and *At1g51890* (lower panel) in seedlings of Col-0, *bak1-4* and *bak1-5* treated with 100 nM flg22 or 100 nM elf18 was measured by qPCR analysis. Results are average ± s.e. (n = 3). D. *bak1-5* is hyper-susceptible to *Pseudomonas syringae* pv. *tomato* (*Pto*) DC3000 *COR^-^*. Four-week old plants (Col-0, *fls2*, *efr*, *fls2 efr*, *bak1-4 and bak1-5*) were spray-inoculated and bacterial count measured 3 d.p.i.. Results are average ± s.e. (n = 4). “a”, “b”, or “c” above the graph denotes statistically significant difference p<0.05 (ANOVA, Newman-Kleus post test). These experiments were repeated at least three times with similar results.

We found that the ROS burst induced by flg22 and elf18 treatment was strongly reduced in *bak1-5* leaves (Figure 2A), whereas leaves of the null mutant *bak1-4* showed only a delayed and slightly reduced ROS burst (Figure 2A), as previously reported [26], [27].

Next, we analysed the impact of *bak1-5* on the activation of MAP kinases (MPKs) by flg22 and elf18. Consistent with previous observations, the activation of MPK3, 4 and 6 after flg22 and elf18 treatment was delayed and reduced in *bak1-4* seedlings (Figure 2B). Surprisingly, the activation of these MPKs by flg22 and elf18 was differentially regulated in *bak1-5* seedlings. The activation of MPK3 and 6 by flg22 and elf18 was also delayed, but the level of activation ultimately reached levels similar to that observed in wild-type seedlings at 15 mins. Notably, MPK4 was not activated at all during the time-course of the experiment (Figure 2B).

Since MPK activation is linked to PAMP-induced transcriptional reprogramming [37], [38] we then assessed whether PAMP-induced gene expression was also affected in *bak1-5* seedlings using three different PTI marker genes [39] over a 3-hour time-course experiment. The induction of the three genes by flg22 and elf18 was partially impaired in *bak1-4* over the time-course although this effect was minor at certain time-points (Figure 2C). In contrast, after flg22 or elf18 treatment the transcript levels of all three PTI-marker genes were drastically reduced in *bak1-5* over the time-course (Figure 2C). Interestingly, the steady-state expression of the marker genes was already significantly lower in *bak1-5* when compared to wild-type (Figure S3).

Our results clearly demonstrate that *bak1-5* plants were strongly affected in all flg22 and elf18 responses measured. Strikingly, the new allele *bak1-5* was more strongly impaired in PTI signaling than the null allele *bak1-4* suggesting a mis-regulation of PTI signaling. This effect was particularly apparent with EFR-dependent responses, as *bak1-4* null mutants were not affected in elf18-triggered late responses, whereas *bak1-5* mutants were.

Finally, we tested if the strong impairment of *bak1-5* in EFR- and FLS2-dependent PTI signaling compromised resistance to bacterial pathogen. For this purpose we spray-infected four week-old plants with the weakly virulent strain *Pto* DC3000 *COR^−^* that has been previously shown to be compromised in fully suppressing PTI signaling [40]. Consistently, bacteria grew to slightly higher titters in leaves of PRR single mutants *fls2* or *efr*, and to even higher levels in the double mutant *fls2 efr* when compared to wild-type (Figure 2D). As reported previously [23], *bak1-4* mutants were as susceptible as wild-type to bacterial spray-infection (Figure 2D); most likely due to the only slight impairment in PTI signaling and the compromised cell death control [23]. In contrast, *bak1-5* plants were hyper-susceptible and supported bacterial multiplication to similar levels as in *efr fls2* leaves (Figure 2D). The impairment of *bak1-5* in bacterial resistance was further supported by the increased disease symptoms observed after spray-infection with *Pto* DC3000 *COR^−^* (Figure S4). In addition, Col-0, *bak1-4* and *bak1-4* plants expressing BAK1 displayed no significant disease symptoms after spray-infection with *Pto* DC3000 *COR^-^*, whereas *bak1-5* or *bak1-4* plants expressing BAK1-5 clearly develop chlorotic lesions associated with disease (Figure S4).

Therefore, the compromised PTI signaling capacity of *bak1-5* leads to a reduced ability to launch effective defence responses culminating in hyper-susceptibility to bacteria.

### *bak1-5* is not impaired in brassinosteroid signaling

**Figure 3:**
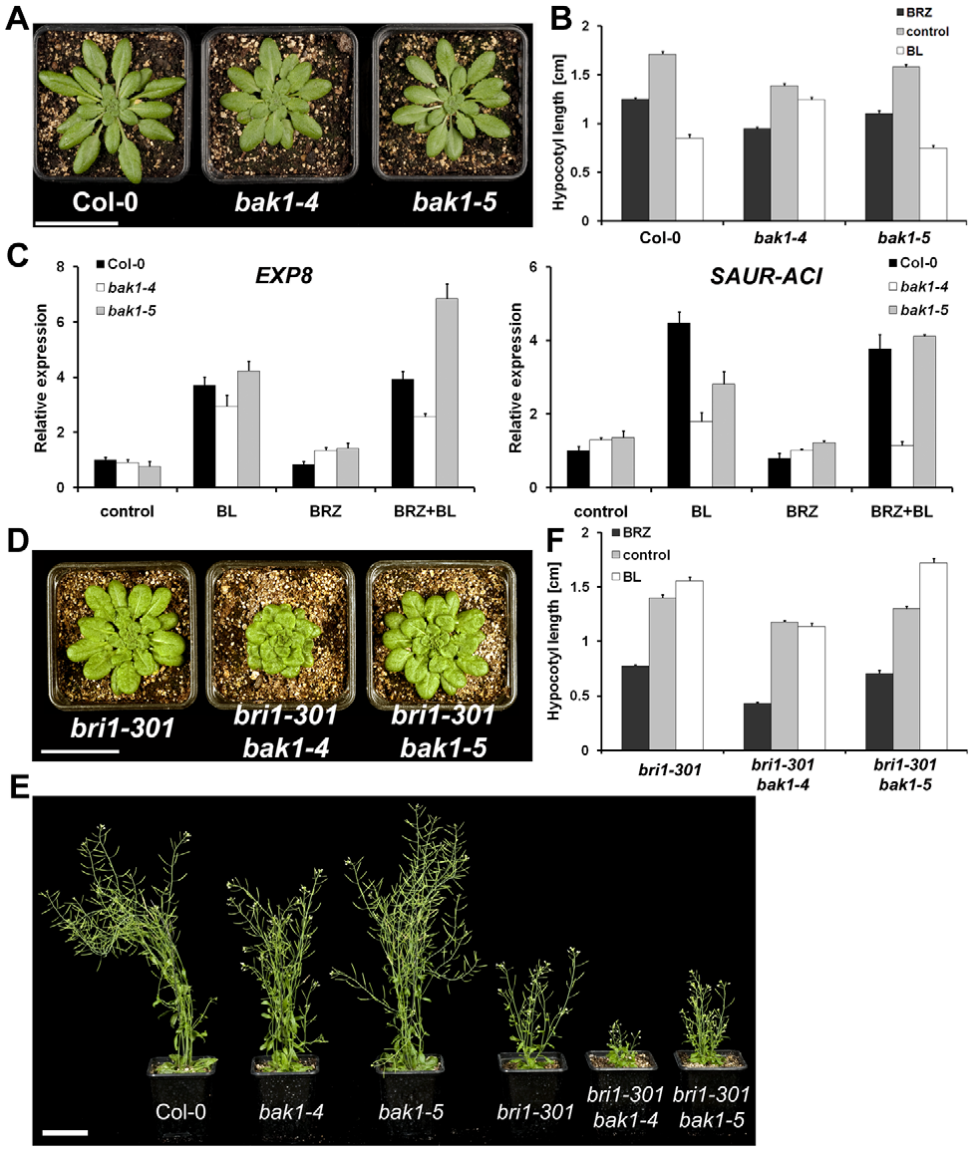
*bak1-5* is not impaired in brassinosteroid signaling. A. *bak1-5* plants have a wild-type-like morphology under short day conditions. Picture of representative individuals of five-week-old Col-0, *bak1-4* and *bak1-5* plants grown under short-day conditions. Scale bar represents 5 cm. B. *bak1-5* shows a wild-type-like BL-induced hypocotyl growth inhibition in etiolated seedlings. Hypocotyl length of 5-day-old etiolated Col-0, *bak1-4* and *bak1-5* seedlings grown without or with 100 nM BRZ or 100 nM BL. Results are average ± s.e. (n≥30). C. *bak1-5* shows a wild-type-like BL marker gene induction. Col-0, *bak1-4* and *bak1-5* seedlings were pre-treated for 16 H with 2.5 mM BRZ or not before treatment with 100 nM BL or not for 3 H. Gene expression of *EXP8* (left) and *SAUR-ACI* (right) was measured by qPCR. Results are average ± S.E. (n = 3). D. *bak1-5* does not aggravate the *bri1-301* cabbage-like rosette under short-day conditions. Picture of representative individuals of six-week-old *bri1-301*, *bri1-301 bak1-4* and *bri1-301 bak1-5* plants grown under short-day conditions. Scale bar represents 5 cm. E. *bak1-5* shows a wild-type-like morphology and does not enhance the *bri1-301* growth phenotype under long-day conditions. Picture of representative individuals of six-week-old Col-0, *bak1-4*, *bak1-5*, *bri1-301*, *bri1-301 bak1-4* and *bri1-301 bak1-5* plants grown under long-day conditions. Scale bar represents 5 cm. F. *bri1-301 bak1-5* is slightly hyper-responsive to BL-induced hypocotyl elongation of etiolated seedlings. Hypocotyl length of 5-day-old *bri1-301*, *bri1-301 bak1-4* and *bri1-301 bak1-5* etiolated seedlings grown without or with 100 nM BRZ or 100 nM BL. Results are average ± s.e. (n≥16). These experiments were repeated at least twice with similar results.

Next, we tested if *bak1-5* was also impaired in BR signaling, as all previously reported *bak1* loss-of-function alleles are hyposensitive to BR [18], [19]. Classically, the reported *bak1* loss-of-function alleles display a semi-dwarf cabbage-like rosette when grown under short-day conditions similar to weak *bri1* mutant plants [18], [19]. Surprisingly, *bak1-5* plants did not show any growth impairment under these conditions and looked comparable to wild-type plants (Figure 3A). Consistently, the expression of both BAK1 and BAK1-5 was able to rescue the semi-dwarf cabbage-like rosette phenotype of *bak1-4* (Figure S5).

As plant morphology does not always correlate with defects in other BR responses [17], we compared the effect of exogenous treatments with brassinolide (BL), the most bioactive BR [41], or the BR biosynthesis inhibitor brassinazole (BRZ) [42] on *bak1-4* and *bak1-5* plants. First, we quantitatively investigated the BR-responsiveness of etiolated seedlings grown under different BR regimes [43]. As expected, *bak1-4* hypocotyls were much smaller than wild-type, were hypo-sensitive to the growth inhibition effect of BL, and hyper-sensitive to BRZ (Figure 3B). In contrast, although *bak1-5* hypocotyls were slightly smaller than wild-type, they displayed a wild-type-like responsiveness to BRZ and BL (Figure 3B).

To test for subtle changes in BR sensitivity in the *bak1-4* and *bak1-5* seedlings, we performed BL marker gene analysis by quantitative real-time RT-PCR. For this purpose, we investigated the expression pattern of two well-characterised BL marker genes, *SAUR-AC1* (*At4g38850*) as an auxin co-regulated gene, and *EXP8* (*At2g40610*) as a BL-specific gene [44]. In order to fully capture the signaling capability of either *bak1* allele, we included a pre-treatment with BRZ to reduce any hormone level adaptation within genotypes that may have altered BR signaling capacity as previously reported for *bzr1-1D* [45]. BL treatment on its own did not reveal any significant differences between the genotypes for *EXP8* expression (Figure 3C, left). However, the induction of *SAUR-AC1* by BL was clearly impaired in *bak1-4* and less so in *bak1-5* (Figure 3C, right). Interestingly, BRZ pre-treatment prior to BL treatment revealed a clear impairment of *bak1-4* in BL-induced gene expression for both marker genes (Figure 3C). On the contrary, *bak1-5* showed an induction of *SAUR-AC1* comparable to wild-type (Figure 3C, right), and the induction of *EXP8* appeared higher in *bak1-5* than wild-type under this treatment regime (Figure 3C, left).

Defects in BR sensitivity are often revealed when mutations in potential BR signaling components or biosynthetic genes are combined with weak *bri1* alleles [46]. To test if the *bak1-5* mutation affects BR sensitivity in such assays, we crossed *bak1-4* or *bak1-5* with *bri1-301* that carries a point mutation in the kinase domain of BRI1 [47]. As previously reported [18], the *bak1-4* mutation increased the BR-related phenotypes of *bri1-301*, as measured by rosette morphology of short-day-grown plants, hypocotyl length of etiolated seedlings grown on BL- or BRZ-containing medium, and morphology of long-day grown plants (Figure 3D–3F). In contrast, the *bak1-5* mutation did not aggravate the *bri1-301* phenotype to the same extent in any of these assays (Figure 3D–3F). Surprisingly, as noted before with the expression of BL marker genes in *bak1-5* (Figure 3C), etiolated *bri1-301 bak1-5* seedlings appeared even slightly hyper-responsive to BL when compared to *bri1-301* (Figure 3E).

Overall, our results clearly demonstrate that the novel allele *bak1-5* is still fully sensitive to BR. This phenotype is in clear contrast with the hypo-sensitivity generally associated with *bak1* loss-of-function alleles.

### *bak1-5* is not impaired in cell death control

**Figure 4:**
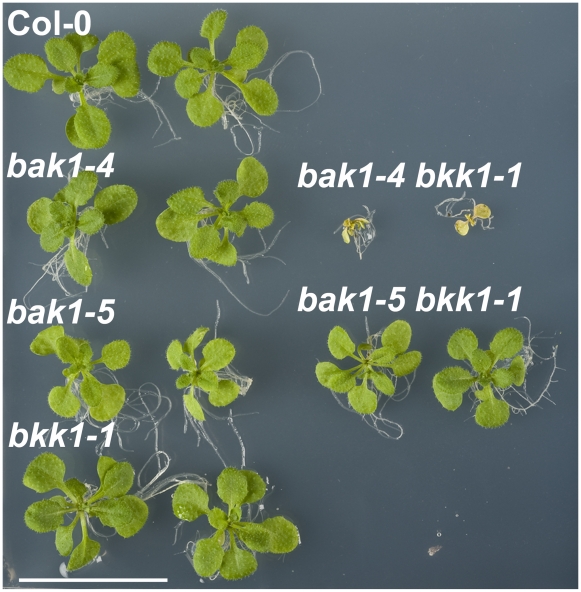
Cell death control is not compromised in *bak1-5.* Picture of representative individuals of 2.5 week-old seedlings of Col-0, *bak1-4*, *bak1-5*, *bkk1-1*, *bak1-4 bkk1-1* and *bak1-5 bkk1-1*. Scale bar represents 2 cm. These experiments were repeated at least three times with similar results.

To test if *bak1-5* is impaired in cell death control, we crossed *bak1-4* or *bak1-5* with the null mutant *bkk1-1* (SALK_057955) [24]. Twenty out of seventy individuals (*X* ^2^ = 0.476, *p* = 0.49) from a *bak1-4×bkk1-1* F2 segregating population died after two weeks in long-day conditions on sterile MS plates. In contrast, none of *bak1-5×bkk1-1* F2 segregating seedlings (n = 76) died, and we could isolate fully viable double mutants (Figure 4). Furthermore, homozygous *bak1-5 bkk1-1* plants showed no symptoms related to cell death or early senescence when grown in non-sterile soil, and this even at later stages of development (Figure S6).

The *bak1-5* allele is therefore not associated with loss of cell death control.

### BAK1-5 shows an enhanced interaction with the ligand-binding LRR-RKs FLS2, EFR, and BRI1

From our detailed phenotypic analysis (Figure 2, Figure 3, Figure 4), it appears that *bak1-5* is specifically affected in PTI signaling. One hypothesis for the observed phenotypes could be that BAK1-5 has a reduced interaction with the PRRs FLS2 and EFR, but is still capable of interacting with the BR receptor BRI1.

**Figure 5:**
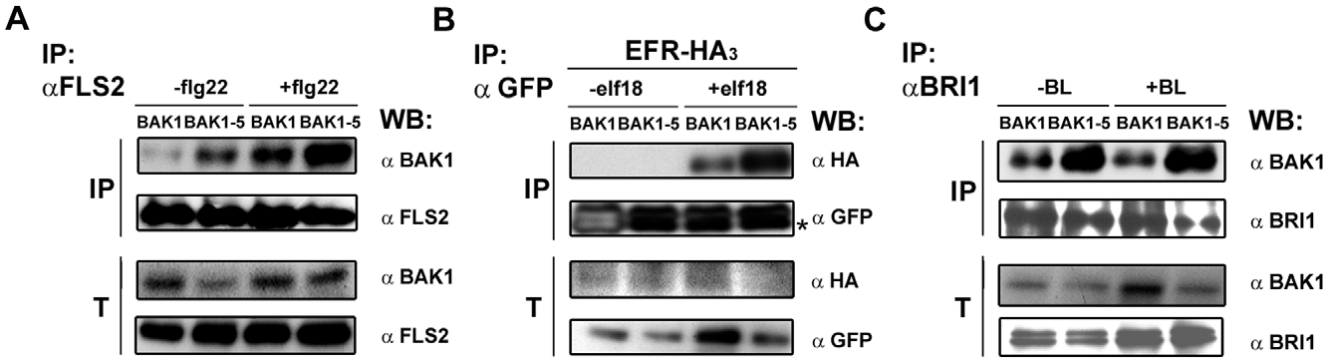
BAK1-5 shows an enhanced interaction with ligand-binding RK FLS2, BRI1, and EFR. A. BAK1-5 shows a ligand-independent interaction with FLS2 in *A. thaliana*. Co-immunoprecipitation of BAK1 or BAK1-5 with FLS2 in Col-0 or *bak1-5* plants treated or not with 100 nM flg22 for 5 min, respectively. Total proteins (T) were subjected to immunoprecipitation (IP) with anti-FLS2 antibodies and IgG beads followed by immunoblot analysis using either anti-FLS2 or anti-BAK1 antibodies. B. BAK1-5-GFP shows an enhanced interaction with EFR-HA_3_ in *N. benthamiana*. Co-immunoprecipitation of leaves expressing EFR-HA_3_ with either BAK1-GFP or BAK1-5-GFP. Leaves were treated or not with 100 nM elf18 for 5 min. Total proteins (T) were subjected to immunoprecipitation (IP) with GFP-Trap beads followed by immunoblot analysis using either anti-GFP or anti-HA antibodies. The asterisk indicates an unspecific band. C. BAK1-5 shows an enhanced interaction with BRI1 in *A. thaliana*. Co-immunoprecipitation of BAK1 or BAK1-5 with BRI1 in Col-0 or *bak1-5* treated or not with 100 nM BL for 1.5 H, respectively. Total proteins (T) were subjected to immunoprecipitation with anti-BRI1 antibodies and IgG beads followed by immunoblot analysis using either anti-BRI1 or anti-BAK1 antibodies. The asterisk indicates an unspecific band. These experiments were repeated at least twice with similar results.

To test this hypothesis, we performed co-immunoprecipitation analyses between BAK1 and these receptors. Using specific anti-FLS2 antibodies, we could detect a clear flg22-dependent complex formation between FLS2 and BAK1 in wild-type Arabidopsis seedlings (Figure 5A). Surprisingly, BAK1-5 was detected in FLS2 immunoprecipitates from non-elicited seedlings (Figure 5A). In addition, the amount of BAK1-5 in complex with FLS2 after flg22 treatment was greater than in the case of BAK1 (Figure 5A). Similar results were observed when we performed the reciprocal immunoprecipitation experiment (Figure S7).

We recently demonstrated that BAK1 also forms a ligand-dependent complex with EFR (Roux et al., *submitted*). Due to the lack of specific anti-EFR antibodies that could be used for immunoprecipitation experiments in Arabidopsis, we tested the interaction of epitope-tagged BAK1 or BAK1-5 with EFR after heterologous transient expression in the plant model *N. benthamiana*. After immunoprecipitation of BAK1-GFP using GFP-trap beads we observed a clear elf18-dependent recruitment of EFR-HA_3_ into the complex (Figure 5B). Interestingly, the amount of EFR-HA_3_ present with BAK1-5-GFP in complex after elf18 treatment was higher than with BAK1-GFP (Figure 5B).

Next, we tested the interaction of BAK1-5 with BRI1 after immunoprecipitation with specific anti-BRI1 antibodies (Figure 5C). We were able to confirm the *in planta* BRI1-BAK1 interaction previously reported using transgenic lines expressing epitope-tagged BRI1 and/or BAK1 proteins [18], [20]. Surprisingly, as observed with FLS2 and EFR, BAK1-5 also showed an enhanced interaction with BRI1 (Figure 5C).

Importantly, BAK1-5 still retained its interaction specificity, as it did not interact with CERK1, a LysM-RK involved in BAK1-independent chitin perception [29], [48], [49], when co-expressed as epitope-tagged proteins in *N. benthamiana* (Figure S8).

In contrast to our initial hypothesis, BAK1-5 has a higher affinity than BAK1 for the ligand-binding LRR-RKs FLS2, EFR and BRI1. This observation, together with the differential impact of the *bak1-5* mutation on PTI signaling triggered by FLS2 and EFR, but not on BRI1-dependent responses (Figure 2 and Figure 3), indicates that the *bak1-5* phenotype cannot be solely explained by differences in complex formation.

### BAK1-5 is a hypoactive kinase

Since the *bak1-5* mutation corresponds to a C408Y amino acid change just before the catalytic loop of the kinase domain (Figure 1C), the *bak1-5* phenotype could be due to altered kinase activity.

**Figure 6:**
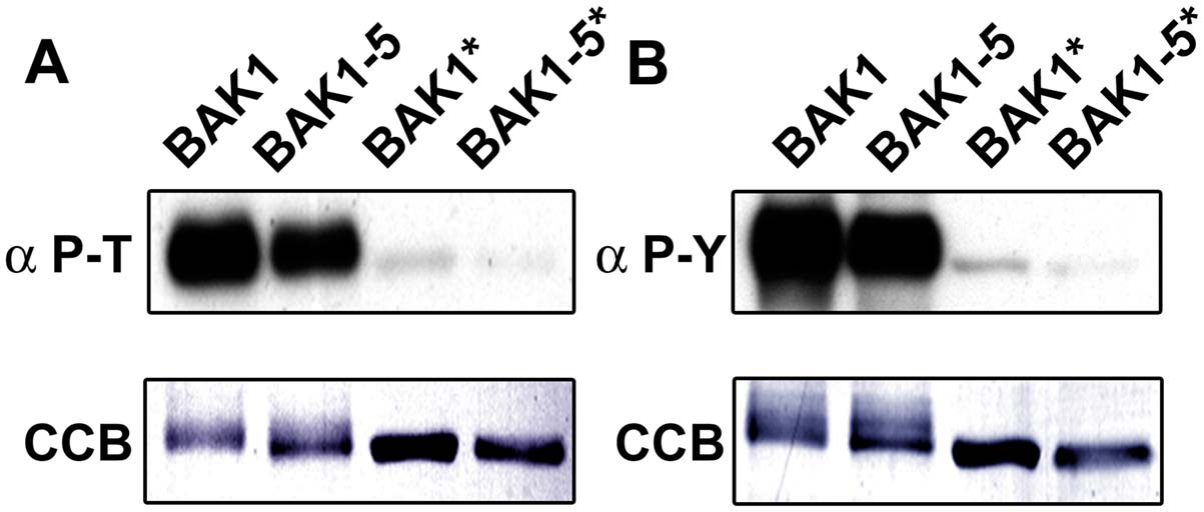
BAK1-5 is a hypoactive kinase *in vitro.* A. BAK1-5 CD is a hypoactive kinase on Ser and Thr residues. 0.25 µg of heterologously-expressed N-terminal GST-tagged BAK1, BAK1-5, BAK1* and BAK1-5* CD were subjected to immunoblot analysis with anti-phospho-Thr antibodies. Immunoblot, upper panel; Coomassie colloidal blue stained membrane, lower panel. B. BAK1-5 CD is a hypoactive kinase on Tyr residues. 0.75 µg of heterologously-expressed N-terminal GST-tagged BAK1, BAK1-5, BAK1* and BAK1-5* CD were subjected to immunoblot analysis with anti-phospho-Tyr antibodies. Immunoblot, upper panel; Coomassie colloidal blue stained membrane, lower panel. These experiments were repeated at least twice with similar results.

To test potential differences in BAK1-5 kinase activity, we expressed in *Escherichia coli* (*E. coli*) the cytoplasmic domains (CD: residues 256 to 615) of BAK1 and BAK1-5, as well as the respective kinase-dead mutant variants (D416N) (indicated as BAK1* and BAK1-5*, respectively) as N-terminally tagged GST-fusion proteins and purified them using glutathione beads. In agreement with previous studies [18]–[21] we detected a strong phosphorylation of BAK1 CD on threonine/serine and tyrosine residues *in vitro* (Figure 6A–6B). This is due to the auto-phosphorylation of BAK1 CD during recombinant protein production and in the *in vitro* kinase assay as the phosphorylation status of kinase dead BAK1* CD was negligible (Figure 6A–6B). The phosphorylation status of BAK1-5 CD was slightly reduced compared to BAK1 CD but still significantly higher than that of kinase dead BAK1-5* CD (Figure 6A–6B). This is also illustrated by the fact that both BAK1 CD and BAK1-5 CD showed a mobility shift on SDS-PAGE compared to kinase inactive mutant variants (Figure 6A–6B). Next, we quantified the reduction of kinase activity of BAK1-5 by determining the auto-phosphorylation levels of BAK1 and BAK1-5 over an increasing concentration range of ATP. As shown in Figure S9, BAK1-5 has an ∼3.6-fold reduction in kinase activity as the C408Y mutation in BAK1-5 lowers its K_m_ to ∼25 µM compared to ∼7 µM in the case of BAK1. These results demonstrate that BAK1-5 is an active kinase albeit with a slightly reduced kinase activity when compared to BAK1.

### The RD kinases BRI1 and BAK1 differ from the non-RD kinases EFR and FLS2 in their phosphorylation activities

BAK1 CD and BRI1 CD are active kinases that undergo auto- and trans-phosphorylation when incubated together *in vitro* [18], [19], [21]. Therefore, we studied the kinase activities of FLS2 CD and EFR CD, and the trans-phosphorylation events occurring between them and the BAK1 CD.

**Figure 7:**
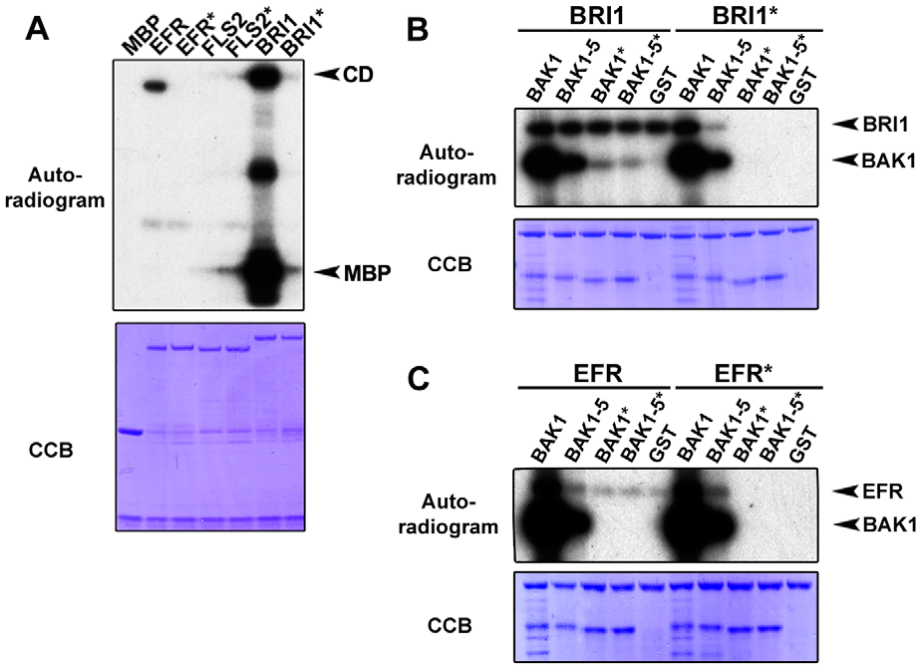
Differential phosphorylation activity of BRI1 and BAK1 (RD-kinases) and FLS2 and EFR (non-RD kinases). A. Differential kinase activity of the RD kinase BRI1 and the non-RD kinases FLS2 and EFR. *In vitro* kinase assay incubating equal amounts of MBP control or N-terminal MBP-tagged EFR, EFR*, FLS2, FLS2*, BRI1 and BRI1* CD with artificial substrate myelin basic protein (MBP). Autoradiogram, upper panel; Coomassie colloidal blue stained membrane, lower panel. B. BRI1 and BAK1 undergo bi-directional trans-phosphorylation *in vitro*. *In vitro* kinase assay incubating equal amounts of N-terminal MBP-tagged BRI1 or BRI1* CD with N-terminal GST-tagged BAK1, BAK1*, BAK1-5, BAK1-5* CD or GST control, respectively. Autoradiogram, upper panel; Coomassie colloidal blue stained membrane, lower panel. C. Uni-directional trans-phosphorylation of EFR by BAK1 *in vitro*. *In vitro* kinase assay incubating equal amounts of N-terminal MBP-tagged EFR or EFR* CD with N-terminal GST-tagged BAK1, BAK1*, BAK1-5, BAK1-5* CD or GST control, respectively. Autoradiogram, upper panel; Coomassie colloidal blue stained membrane, lower panel. These experiments were repeated at least three times with similar results.

We first analyzed FLS2 and EFR kinase activities and compared them with the kinase activity of BRI1. For this purpose, we expressed in *E. coli* the CDs of EFR (residues 682 to 1031), FLS2 (residues 840 to 1173) and BRI1 (residue 814 to 1196) as fusion proteins with an N-terminal maltose-binding protein (MBP) tag. As controls, we also constructed the respective kinase-dead variants EFR* CD (D849N), FLS2* CD (D997N) and BRI1* CD (D1009N). We initially intended to identify the phosphorylation status of FLS2 CD, EFR CD and BRI1 CD using phospho-site specific antibodies either recognizing phosphorylated threonine/serine or tyrosine residues. Unfortunately, we were unable to observe a signal specific to the kinase active variants of FLS2 CD and EFR CD (data not shown), therefore we restored to using radioactive [^32^P]-γ-ATP in *in vitro* kinase assays. As previously reported [50], BRI1 CD had a very strong auto- and trans-phosphorylation capacity using the artificial substrate myelin basic protein (MBP) (Figure 7A). In contrast, EFR CD possessed only minor auto-phosphorylation capacity and negligible trans-phosphorylation ability on MBP (Figure 7A). Notably, these activities were abolished in BRI1* CD and EFR* CD (Figure 7A), demonstrating that the observed phosphorylations are indeed due to the intrinsic kinase activities of these protein.

Surprisingly, we were unable to detect any FLS2 CD phosphorylation *in vitro* (Figure 7A), indicating that FLS2 is an extremely weak kinase. The latter result is in disagreement with previous reports that revealed phosphorylation activities *in vitro* for FLS2 [31], [50], [51]. As Zhou and colleagues [31] used a N-terminally His tagged FLS2 fusion protein to report FLS2 kinase activity, we also generated His-FLS2 CD. Again, as observed with MBP-FLS2 CD, we were unable to observe any phosphorylation activity (Figure S10). Under the same conditions, His-BRI1 CD displayed a strong kinase activity (Figure S10).

Consequently, it appears that in comparison BRI1 is an extremely strong kinase, EFR is a moderately good kinase, and FLS2 is almost kinase-inactive *in vitro*. Therefore, we focused our trans-phosphorylation studies with BAK1 and BAK1-5 on the comparison between the non-RD kinase EFR and the RD kinase BRI1.

We first confirmed in our experimental conditions that BAK1 CD was able to trans-phosphorylate BRI1* CD, and reciprocally that BRI1 CD was able to trans-phosphorylate BAK1* CD (Figure 7B). Also BAK1-5* CD was trans-phosphorylated by BRI1 CD and to a similar level compared to BAK1* CD (Figure 7B). The reduced kinase activity of BAK1-5 CD lead to a lower level of trans-phosphorylation of BRI1* CD when compared to BAK1 CD (Figure 7B).

Next, we investigated the *in vitro* trans-phosphorylation events surrounding EFR CD. We found that BAK1 CD was able to trans-phosphorylate EFR* CD to a level much stronger than EFR CD auto-phosphorylation (Figure 7C). This is in contrast to the BAK1-BRI1 trans-phosphorylation events in which BAK1 CD trans-phosphorylation of BRI1* CD is similar in comparison to BRI1 CD auto-phosphorylation (Figure 7B). Another striking difference was the inability of EFR CD to trans-phosphorylate BAK1* CD (Figure 7C). Importantly, BAK1-5 CD was still able to trans-phosphorylate EFR* CD and slightly enhanced the phosphorylation status of EFR CD (Figure 7C).

In summary, BAK1 trans-phosphorylates the non-RD kinase EFR, but not the reverse. In contrast, the RD-kinase BRI1 undergoes a bi-directional trans-phosphorylation with BAK1 *in vitro* as previously shown [19], [21]. This is particularly interesting as BAK1-5 displays a reduced trans-phosphorylation capacity for both receptors *in vitro* but specifically blocks signaling events mediated by the non-RD kinase EFR *in vivo* (Figure 2 and Figure 3).

### Kinase activitiy is not required for ligand-dependent FLS2/EFR-BAK1 heteromerization

The kinase activity of BRI1 is strictly required for the ligand-induced BRI1-BAK1 complex formation [21]. To determine whether the *in vivo* heteromerization of BAK1 with FLS2 or EFR requires the kinase activity of either partner, we co-expressed in *N. benthamiana* wild-type and kinase-dead versions of FLS2, EFR and BAK1 for co-immunoprecipitation experiments.

**Figure 8:**
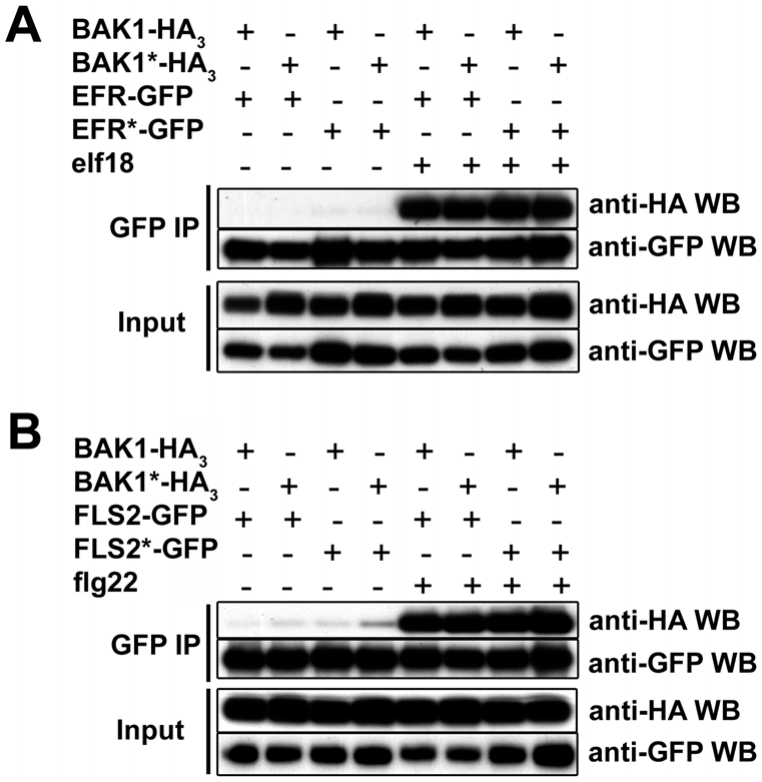
The ligand-induced heteromerization of EFR and FLS2 with BAK1 is independent of kinase activity *in planta*. A. Elf18-induced co-immunoprecipitation of EFR and BAK1 before (−) and after (+) elicitation with 100 nM elf18 in *N. benthamiana* transiently expressing EFR-GFP-His or EFR*-GFP-His and BAK1-HA_3_ or BAK1*-HA_3_, as indicated. Total proteins were subjected to immunoprecipitation with anti-GFP beads followed by immunoblot analysis with anti-GFP or anti-HA antibodies as indicated. B. Flg22-induced co-immunoprecipitation of FLS2 and BAK1 before (−) and after (+) elicitation with 100 nM flg22 in *N. benthamiana* transiently expressing FLS2-GFP-His or FLS2*-GFP-His and BAK1-HA_3_ or BAK1*-HA_3_, as indicated. Total proteins were subjected to immunoprecipitation with anti-GFP beads followed by immunoblot analysis with anti-GFP or anti-HA antibodies as indicated. These experiments were repeated twice with similar results.

Clear ligand-dependent complex formation between the wild-type BAK1 and FLS2 or EFR proteins could be detected (Figure 8A–8B). Co-expressing BAK1*-HA_3_ with either FLS2-GFP or EFR-GFP did not reduce the complex formation after PAMP treatment when immunoprecipitating FLS2 or EFR using GFP-trap beads (Figure 8). Similarly FLS2*-GFP and EFR*-GFP possessed full interaction capacity after ligand addition when co-expressed with BAK1-HA_3_ (Figure 8A–8B). Finally, we tested double kinase-dead receptor combinations. After ligand addition, both FLS2*-GFP and EFR*-GFP still interacted with BAK1*-HA_3_ as strongly as wild-type receptor combinations (Figure 8A–8B).

Thus, the kinase activities of neither FLS2/EFR nor BAK1 are required for their ligand-induced heteromerization.

### The kinase activity of BAK1-5 is required for the *bak1-5* phenotype

We tested if the kinase activity of BAK1-5 is required for the *bak1-5* phenotype. As *bak1-5* has the strongest differential phenotype with elf18 response when compared to *bak1-4*, we concentrated on EFR-dependent responses to address this question.

**Figure 9:**
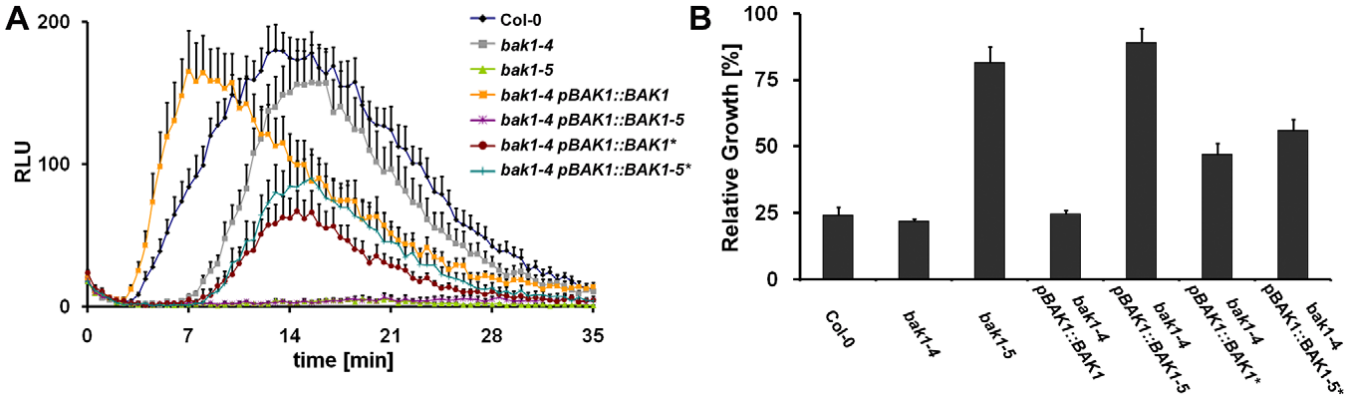
BAK1-5 requires its kinase activity for suppression of elf18-induced responses. A. The kinase activity of BAK1-5 is required for the suppression of elf18-induced ROS burst. ROS burst in leaves of Col-0, *bak1-4*, *bak1-5*, *bak1-4 pBAK1::BAK1*, *bak1-4 pBAK1::BAK1-5 bak1-4*, pBAK1::BAK1* and bak1-4 pBAK1::BAK1-5* treated with 100 nM elf18. Results are average ± s.e. (n = 8). B. The kinase activity of BAK1-5 is required for the suppression of elf18-induced SGI. SGI of Col-0, *bak1-4*, *bak1-5*, *bak1-4 pBAK1::BAK1*, *bak1-4 pBAK1::BAK1-5 bak1-4*, pBAK1::BAK1* and bak1-4 pBAK1::BAK1-5* in the presence of 60 nM elf18. Results are average ± s.e. (n = 6). These experiments were repeated at least twice with similar results.

We created stable transgenic lines in the *bak1-4* background expressing BAK1, BAK1*, BAK1-5 and BAK1-5* under the native regulatory sequence of *BAK1* (Figure S11). The wild-type allele of BAK1 was able to rescue the reduced and delayed elf18-induced ROS burst of *bak1-4* (Figure 9A). As previously shown (Figure 1E), expression of BAK1-5 in *bak1-4* recapitulated the *bak1-5* phenotype (Figure 9B). Interestingly, the expression of the kinase inactive BAK1* in *bak1-4* led to a further decrease in elf18-induced ROS burst (Figure 9B), revealing a dominant-negative effect of BAK1* and demonstrating the importance of BAK1 kinase activity for downstream signaling. Strikingly, the expression of BAK1-5* in *bak1-4* led to a similar dominant-negative effect as BAK1* but did not fully suppress elf18-induced ROS burst as observed in *bak1-5* or when BAK1-5 was expressed in *bak1-4* (Figure 9A). Similar results were observed in the SGI assay (Figure 9B).

These two observations demonstrate that BAK1-5 requires its kinase activity to quench EFR-dependent signaling. More importantly, it strongly suggests that the differential impact of the *bak1-5* mutation on different signaling pathways is linked to phosphorylation.

## Discussion

Plants need to correctly process diverse exogenous and endogenous information. For this purpose they rely heavily on surface localised ligand-binding RKs and regulatory RLKs. In recent years, the importance of the regulatory RLK BAK1 became apparent, as it is involved in several independent signaling pathways, namely BR responses, innate immunity and cell death control [14]. It was however unclear whether the regulatory role and the importance of BAK1 in these different biological processes are similar. Here, we clearly demonstrated that BAK1 differentially regulates these pathways in a phosphorylation-dependent manner.

### Phosphorylation-dependent differential regulation of BAK1-dependent BR and PTI signaling pathways

We found that *bak1-5* mutant plants are impaired in all early and late elf18- and flg22-triggered responses tested (Figure 1 and Figure 2). Yet, *bak1-5* possesses full signaling capacity for BR signaling (Figure 3). This is in contrast with previously described *bak1* loss-of-function mutant alleles that are partially impaired in early and late flg22-triggered responses, but only in early responses triggered by elf18 (Figure 2; Figure S1) [26], [27]. Importantly, *bak1* loss-of-function mutants are also weakly impaired in BR signaling (Figure 3; Figure S5) [18], [19]. Our initial working hypothesis for the differential regulation of BR and PTI signaling in *bak1-5* was based on a potential differential interaction of BAK1-5 with the different ligand-binding RKs. However, this simple hypothesis did not hold true as BAK1-5 displays an enhanced interaction with all three ligand-binding RKs tested, namely FLS2, EFR and BRI1 (Figure 5). Therefore, we investigated the kinase activity of BAK1-5 and were able to show that BAK1-5 possesses considerable kinase activity albeit slightly reduced compared to BAK1. Importantly, BAK1-5 was still able to trans-phosphorylate both BRI1 and EFR *in vitro* (Figure 7B–7D). This raises the alternative hypothesis that the reduced kinase activity of BAK1-5 is sufficient to support BR but not PTI signaling. Yet several observations do not support this hypothesis. First, there is no direct correlation between the *in vitro* kinase activity of BAK1 mutant variants and their ability to complement either the compromised flg22-triggered SGI of *bak1-4 bkk1-1* or the growth retardation phenotype of *bri1-5* [21]. BAK1(T449A) is able to complement both phenotypes but has a reduced kinase activity compared to BAK1(T450A) that is not able to complement either phenotype [21]. Interestingly, BAK1-5 possesses a stronger kinase activity than BAK1(T449A) (data not shown) further substantiating this observation. Second, plants expressing the hypo-active kinase variant BAK1(Y610F) are blocked only in BR signaling but not flg22-triggered SGI [34] thereby displaying an opposite phenotype to *bak1-5* plants even though both BAK1 variants are compromised in their overall kinase activity. Therefore, the quantitative kinase output of BAK1 is not the determining factor *per se* that enables BAK1 to function in PTI- or BR-signaling (Table S1). Third, in *bak1-5* plants PTI signaling is not simply more impaired than in *bak1-4* loss of function mutants but rather differentially regulated. This is exemplified in the differential MPK activation in *bak1-5* plants whereby MPK3 and 6 but not MPK4 are fully activated 15 mins after ligand-treatment (Figure 2B). Fourth, BAK1-5 requires its kinase activity to fully suppress elf18-triggered ROS-burst *in vivo* (Figure 9B).

Altogether, this leads to the new hypothesis that BAK1-5 differentially regulates PTI- and BR-signaling pathways by discriminative auto-phosphorylation and/or trans-phosphorylation of the main-ligand binding receptors. Therefore, the qualitative kinase output of BAK1 defines its signal competence in respect to PTI- or BR- signaling pathways.

In the case of *bak1-5* mutant plants, the differential auto-phosphorylation of BAK1-5 could theoretically already lead to a differential interaction surface for potential downstream signaling components. Alternatively (or concomitantly), BAK1-5 could trans-phosphorylate specific residues on EFR and FLS2 that would affect interactions with positive and/or negative regulators, such as BIK1 and related proteins [50], [52]. Phosphorylation of specific phosphosites in the intra-cellular juxta-membrane region and C-terminal tail of mammalian RTKs and Ser/Thr RKs are known to regulate signal complex composition, sub-cellular localization, receptor degradation, and therefore the initiation, amplitude, complexity and/or duration of the signal [53], . Interestingly, the rice PRR XA21 also seems to be under phosphorylation-dependent negative regulation. The ATPase XB24 interacts with XA21 *in vivo*, promotes XA21 auto-phosphorylation *in vitro*, and is a negative regulator of XA21-mediated immunity [55].

As observed previously for *bak1* loss-of-function [23] and *bak1-4 bkk1-1* plants expressing the phosphosite mutant variant BAK1(Y610F) [34], the basal expression level of several defence marker genes was significantly reduced in *bak1-5* (Figure S3). Since BAK1-5 showed a reduced Tyr phosphorylation level *in vitro* (Figure 6B) BAK1-5 may be unable to auto-phosphorylate on Y610 and that BAK1 normally regulates basal gene expression via phosphorylation of this specific amino acid. Alternatively, the overall reduced kinase activity of both BAK1-5 and BAK1(Y610F) may lead to a lower constitutive basal defence signaling either induced by epiphytic bacteria and/or caused by spontaneous kinase activity [53].

### Regulation of BAK1-dependent cell death control

*bak1-5* is not impaired in cell death control, as *bak1-5 bkk1-1* double mutant is viable and do not show any cell death or early senescence phenotypes (Figure 4 and Figure S6). This peculiarity is currently difficult to interpret, as the role of BAK1 and BKK1 in inducible and constitutive cell death control is still unclear. It was initially speculated that BAK1 and BKK1 might negatively control a ligand-binding RK perceiving a potential endogenous “survival” ligand [23], . Another LRR-RLK, BIR1, interacts with BAK1 *in vivo* and is strictly required for cell death control [25]. An alternative model is suggested by the constitutive cell death phenotype of *bak1-4 bkk1-1* seedlings that is partially dependent on salicylic acid [24], is light-dependent [56], and the fact that the *bir1-1* cell death phenotype is partially reverted by high temperatures and mutations in *PAD4* and *EDS1* [25], components classically associated with R protein-mediated hyper-sensitive response [57]. The integrity and/or activity of a multimeric complex containing BAK1, BKK1 and BIR1 may be “guarded” by an R protein. The absence of BAK1 and BKK1, or BIR1, would thus trigger constitutive cell death and explain the mutant seedling lethality even in sterile conditions. Interestingly though, the kinase activity of BAK1 seems to be important for cell death control, as kinase-dead variants of BAK1 cannot rescue the *bak1-4 bkk1-1* lethality [21]. In this respect, it is not surprising that *bak1-5 bkk1-1* is fully viable as only kinase inactive variants of BAK1 were previously shown to be unable to complement the *bak1-4 bkk1-1* lethality phenotype [21], [34].

### Differential regulation of RD and non-RD kinases

The differential impact of *bak1-5* on BRI1-dependent and FLS2/EFR-dependent signaling could also be related to a more general differential regulation of RD versus non-RD kinases. RD kinases carry an arginine (Arg) before the conserved catalytic core Asp, and generally are activated by phosphorylation in the activation loop. The phospho-groups interact with a positively-charged pocket containing the Arg and most likely re-orient residues within the catalytic loop, ATP-binding pocket and/or facilitate peptide substrate binding [13]. In contrast, non-RD kinases do not require phosphorylation of the activation loop to adopt an active confirmation. They are regulated by different mechanisms such as relief of auto-inhibition by C-terminal extensions [58], Tyr phosphorylation in the P+1 loop [59], or are constitutively active kinases [60]. In several cases the kinase activity of non-RD kinases was shown to be at least partially dispensable for some of their functions [35], [61] suggesting a role as scaffolds. However, EFR and FLS2 require kinase activity for signaling, which implies that they do not function solely as scaffolding proteins.

The RD-kinase BRI1 was far more active *in vitro* in our conditions than the non-RD kinases EFR and FLS2 showing strong auto- and trans-phosphorylation capacities (Figure 7A–7B). EFR did possess some degree of auto-phosphorylation (Figure 7A, 7C), but no trans-phosphorylation capacity either towards the artificial kinase substrate MBP (Figure 7A), or towards the physiologically-relevant BAK1 kinase domain (Figure 7C). Surprisingly, we were unable to detect any *in vitro* activity for FLS2 CD (residues 840 to 1173) neither as N-terminal MBP-tag nor His-tag fusion protein, especially in comparison to the strong BRI1 kinase activity (Figure 7 and Figure S10). This is in contradiction to previous observations that report kinase activity of FLS2 *in vitro* [31], [50], [51], but is in agreement with recent publications reporting only residual kinase activity of FLS2 CD (residues 832 to 1173) and stating that recombinant FLS2 possess only weak kinase activity impeding analysis of trans-phoshorylation events *in vitro* [52]. Notably, close sequence analysis of the FLS2 kinase domain revealed a low conservation of the otherwise highly conserved Gly-rich loop [GxGxxG] in subdomain I, which is involved in the correct positioning of the substrate ATP [62]. Particularly, the replacement of the second invariant Gly by a Ser (S879) in FLS2 is predicted to lead to a dramatic reduction in kinase activity, as mutation of the corresponding Gly in the model Ser/Thr kinase cAPK reduces the kinase activity by 50-fold [63].

In contrast to the situation with BRI1 and BAK1, no trans-phosphorylation of BAK1 by EFR could be observed *in vitro* (Figure 7B–7C). Yet, BAK1 is capable of trans-phosphorylating EFR *in vitro* (Figure 7C). Of course, we cannot exclude that FLS2 and EFR kinase domains are only fully activated *in vivo* after extracellular ligand binding via conformation changes mediated by the trans-membrane domain, which is missing in the *in vitro* system.

Consistent with their low activity *in vitro*, so far no phosphosites could be identified by mass spectrometry on recombinant EFR or FLS2 CDs, even when co-incubated with BAK1 (data not shown). Even in the case of the well-studied non-RD kinase XA21, all studied phosphorylation sites were initially found by targeted mutagenesis and not by mass spectrometry analysis [64], [65]. The identification of specific phosphosites underlying the positive or negative regulation of EFR and FLS2 therefore remain a real technical challenge.

Nevertheless, the kinase activities and some potential phosphosites of FLS2 and EFR are important for several downstream signaling events. A kinase-dead version of EFR (EFR*) is unable to confer elf18-triggered ROS burst when transiently expressed in *N. benthamiana* (Figure S12). A K898M mutation in the FLS2 kinase domain abolished MPK3 and MPK6 activation by flg22 after transient over-expression in *fls2* mutant protoplasts [66]. Targeted mutagenesis of potential phosphosites in FLS2 revealed that T867, T1040 and T1072 are required for its full functionality [67]. However, it was not investigated if these sites are required for kinase activity, are auto-phosphorylation sites, or whether they represent trans-phophorylation targets of BAK1.

Overall, the striking difference between the kinase activities of the two RD kinases BRI1 and BAK1 compared to the non-RD kinases EFR and FLS2 suggests a different regulatory mechanism between these two kinase classes. A highly conserved Thr residue in the intracellular juxta-membrane domain reveals a differential regulation of the overall kinase activity of RD and non-RD kinase by a single site. Accordingly, T705 of the non-RD kinase XA21 is essential for *in vitro* auto-phosphorylation, interaction with downstream signaling components, and for XA21-mediated resistance [65]. Similarly, a mutation of the corresponding residue in FLS2 (T867) compromised its function *in planta* [67]. However, in the case of the RD kinase BRI1 the phosphorylation of the corresponding Thr (T880) is not required for its function [20]. These results suggest that the regulation of auto-phosphorylation of non-RD kinases by phosphosites in the intra-cellular juxta-membrane region may play an important role in the recruitment of downstream signaling components, as suggested in [65].

Another difference between RD and non-RD RK seems to be the requirement of kinase activity for complex formation with the RD-RLK BAK1. We found that the kinase activity of neither interaction partner is required for the ligand-induced interaction of FLS2 or EFR with BAK1 (Figure 8). Optimal ligand-dependent heteromerization could even be induced between double mutant combinations of FLS2* or EFR* with BAK1* (Figure 8). These results obtained after transient over-expression in *N. benthamiana* nicely complement previous pharmacological studies in *A. thaliana* cell cultures [30]. Treatment of cell cultures with the broad-range kinase inhibitor K252a did not block FLS2-BAK1 complex formation, but totally inhibited phosphorylation of either of the interaction partners. Ligand-dependent conformational changes thus seem sufficient to trigger heteromerization between the non-RD kinases EFR and FLS2 with BAK1. Therefore, the interaction of EFR and FLS2 with BAK1 is a requirement rather than a consequence of detectable phosphorylation. This situation is in stark contrast with the absolute requirement of the BRI1 kinase activity for the ligand-induced complex formation with BAK1 *in planta* [21].

### Conclusions

BAK1 is able to dictate specificity of downstream signaling as BAK1-5 nearly totally blocked FLS2- and EFR-mediated PTI signaling but barely influenced cell death control and BRI1-mediated BR signaling. Based on these results and the recent work from Schulze and colleagues [30], we propose a model for the mechanisms underlying EFR/FLS2 heteromerization with BAK1, and the role of BAK1 in the establishment of PTI signaling. EFR and FLS2 most likely exist in close proximity with BAK1 at the plasma membrane in loose pre-formed complexes due to their near instantaneous oligomerization after ligand binding [30]. Conformational changes triggered by ligand binding lead to the stabilization of the complex. This interaction is kinase-independent, but may lead to the activation of the EFR/FLS2 kinase activity by BAK1 via trans-phosphorylation events. Phosphorylation of specific residues on EFR/FLS2 and/or BAK1 leads to the recruitment of downstream signaling components that dictate the specificity of the signaling output. In this model, BAK1 is not a simple enhancer of the kinase activity of the ligand-binding RKs, but is an integral part of the signaling pathway.

Future studies need to carefully address the role of kinase activity of non-RD kinases for PTI signaling and final defence outcomes. Therefore, careful qualitative and quantitative analyses guided by mass-spectrometry of the phosphorylation status of BAK1, BAK1-5, FLS2 and EFR *in vitro* and *in vivo* will shed more light onto the complex regulatory mechanisms of these two model non-RD PRRs by the regulatory RLK BAK1. These studies are however technically challenging, as unlike BRI1, the kinase activity of EFR is very weak and that of FLS2 is practically negligible at least *in vitro*.

## Methods

### Plant material and methods

All mutants and transgenic lines used in this study were in the background of *A. thaliana* ecotype Columbia (Col-0). The Arabidopsis plants were grown on soil or MS salt medium (Duchefa), 1% sucrose and 1% agar with a 10 H or 16 H photoperiod at 20–22°C. The third backcross of *bak1-5* with Col-0 was used for all experiments.

The mutants *bak1-4*, *bkk1-1*, *bri1-301* were previously described [24], [27], [47]. The double mutants *bak1-4 bkk1-1*, *bak1-5 bkk1-1*, *bak1-4 bri1-301*, and *bak1-5 bri1-301* were generated by crossing and genotyped using the primers listed in Table S2.

### *bak1-5* marker design

For *bak1-5* homozygous mutant identification a dCAPS marker was designed using dCAPS Finder 2.0 [68]. The genomic region around the *bak1-5* mutation was PCR amplified using Taq polymerase (Qiagen) and the primers listed in Table S2. The corresponding product was cut with RsaI (NEB) and *bak1-5* derived PCR products contained an additional RsaI site in addition to the internal restriction control site.

### Generation of transgenic plants

The genomic fragment of *BAK1*, including the promoter and the coding region, in *pDONR201* (Invitrogen) was a gift from B. Kemmerling [23]. The corresponding point mutations for BAK1*, *BAK1-5*, and BAK1-5* were introduced by point mutagenesis PCR using primers given in Table S2. The PCR product was digested with 1.5 µl DpnI (NEB) overnight and subsequently transformed into *Escherichia coli* DH5α. The presence of the corresponding mutations and the integrity of the genomic fragments were verified by sequencing. The correct clones were used to transfer the inserts into *pGWB2* [69] using GATEWAY LR CLONASE II enzyme (Invitrogen). The resulting constructs were verified by restriction analysis and electroporated into *Agrobacterium tumefaciens* strain AglI.

All constructs were transformed into Arabidopsis mutant *bak1-4* using the floral dipping method [70]. Transformants were selected on MS agar medium containing 40 µg/ml hygromycin.

### *In vitro* protein analysis

#### Molecular cloning

The kinase domain of *BAK1* in the *pGEMTeasy* vector was a gift from Sacco de Vries [71]. The corresponding point mutations for BAK1*, *BAK1-5*, and BAK1-5* were introduced as described above using primers given in Table S2. The inserts of sequence verified clones were transferred into *pGEX-4T1* using EcoRI and XhoI (NEB) to generate N-terminal GST fusion constructs.

The kinase domain of *BRI1*, *FLS2* and *EFR* were PCR amplified using the primers given in Table S2. The resulting PCR products were cloned either into *pOPINM* or *pOPINF* [72] using the IN-FUSION reagent (Clontech) to obtain N-terminal MBP or His fusion constructs, respectively. The resulting constructs were verified by restriction analysis and sequencing. The corresponding point mutations of BRI1*, FLS2*, and EFR* were obtained as described above using primers given in Table S2.

#### Recombinant protein purification

Recombinant fusion proteins were produced in *E. coli* BL21 (Novagen), extracted using BugBuster reagent (Novagen) containing 1 µl/ml Benzoase (Novagen), 1 KU/ml Lysozyme (Novagen) and 150 µl/ml protease inhibitor cocktail set II (Novagen) and the soluble fraction was used to enrich for fusion proteins. GST-tagged fusion proteins (GST-BAK1, GST-BAK1*, GST-BAK1-5, GST-BAK1-5*) were enriched using Glutathione Sepharose Fast Flow (GE Healthcare) according to the manufactures protocol. MBP-tagged fusion proteins (MBP-BRI1, MBP-BRI1*, MBP-FLS2, MBP-FLS*, MBP-EFR, MBP-EFR*) were enriched using Amylose Resin (NEB) according to manufactures protocol. His-tag fusion proteins (His-BRI1, His-BRI1*, His-EFR, His-EFR*) were enriched using His-Bind Resin (Novagen) according to the manufactures protocol. After elusion fusion proteins were adjusted to the same concentration in 10% glycerol solution and stored at −20°C until usage.

#### In vitro kinase assay

The fusion proteins were incubated in 30 µl kinase buffer (50 mM Tris, pH 7.5, 10 mM MgCl_2_, 10 mM MnCl_2_, 1 mM DTT) in the presence of only 1 µM unlabeled ATP or 1 µM unlabeled ATP and 183 kB of [^32^P]-γ-ATP for 30 min at 30°C with shaking at 900 rpm. The reactions were stopped by adding 2xLDS loading buffer (Invitrogen). The phosphorylation status of fusion proteins was analyzed by audioradiography after separation of one-fourth of the *in vitro* kinase assay by SDS-PAGE followed by western blotting, if not indicated otherwise. In autophosphorylation assays 1 µg fusion protein for MBP- and GST-tagged proteins and 5 µg for His-tagged proteins was incubated with 1 µg of MBP (Fluka). In transphosphorylation assays 1 µg of each fusion protein was used.

For Km determination, *in vitro* kinase assays were performed as previously described [73]. Post electrophoresis, proteins were transferred onto PVDF membranes. Subsequently, the membranes were subjected to autoradiography using a FUJI Film FLA5000 PhosphorImager (Fuji, Tokyo, Japan) to estimate relative activities.

#### Phosphorylation site analysis

The indicated amount of fusion proteins (GST-BAK1, GST-BAK1*, GST-BAK1-5, GST-BAK1-5*) were separated by SDS-PAGE and blotted onto PVDF membrane (Biorad). The immunoblots were blocked in 5% (w/v) BSA (Sigma) in TBS-Tween (0.1%) for 1–2 H. Phospho-Serine/Threonine sites were detected using anti-p-Thr (1∶1000, Cell Signaling Technology) overnight, followed by anti-mouse-HRP conjugated secondary antibodies (1∶5000, Sigma). Phospho-Tyrosine sites were detected using anti-p-Tyr (1∶2000, Cell Signaling Technology) overnight, followed by anti-rabbit-HRP conjugated secondary antibodies (1∶5000, Sigma).

### qRT-PCR

14-days-old seedlings grown for five days on MS plates and than transferred to liquid MS were used for all gene induction studies. RNA was extracted using RNeasy Plant Mini kit (Qiagen) followed by DNase-treatment using Turbo DNA-free (Ambion) and quantified with a Nanodrop spectrophotometer (Thermo scientific). cDNA was synthesized from 2.5 µg total RNA using SuperScript III reverse transcriptase (Invitrogen). SybrGreen master mix (Sigma) was used for qPCR reactions.

For defence gene induction analysis a triplicate of two seedlings each was treated either with water, 100 nM elf18 or 100 nM flg22 for 0, 30, 60 and 180 min and pooled before harvesting. Gene expression of *At2g17740* (*DC1-domain containing protein*), *At5g57220* (*CYP81F2*) and *At1g51890* (*LRR-RLK*) was monitored by qPCR analysis. The expression of each marker gene was normalized to the internal reference gene *At4g05320* (*UBQ10*) and plotted relative to the Col-0 steady-state expression level.

For BR gene expression analysis a triplicate of two seedlings each was treated with either mock solvent control or 2.5 µM BRZ (Sigma) for 16 H over night. The next morning samples were further treated with mock solvent control or 200 nM brassinolide (SRICI) for another three hours before being pooled for harvesting. Gene expression of *At2g40610* (*EXP8*) and *At4g38850* (*SAUR-ACI*) was monitored by qPCR analysis. The expression of each gene was normalized to the internal reference gene *At5g15400* (U-box containing protein) and plotted relative to the Col-0 double mock treated expression level.

### Hypocotyl growth assay

Freshly harvested seeds were surface sterilized and stratified in sterile water at 4°C for 4–6 days in the dark. Individual seeds were put on ½ MS containing 0.8% phytoagar (Duchefa) without hormone, with 100 nM BL or with 100 nM BRZ and left up-right in the dark at 20–22°C. Hypocotyl length was measured after 5-day incubation.

### Bacterial infection assays

The *P. syringae* pv. *tomato* DC3000 *COR^−^* (*Pto* DC3000 *COR^−^*) [40] strain was grown in overnight culture in Kings B medium supplemented with appropriate antibiotics. Cells were harvested by centrifugation and pellets re-suspended in sterile water to OD_600_ = 0.2. Immediately prior to spraying, Silwett L-77 was added to bacteria to a concentration of 0.04% (v/v). Bacteria were sprayed onto leaf surfaces until run-off and plants covered for 3 days. Samples were taken using a cork-borer (2 mm) to cut leaf discs from 2 leaves per plant and 4 plants per genotype. Leaf discs were ground in water, diluted and plated on TSA medium with appropriate selection. Plates were incubated at 28°C and colonies counted 2 days later.

### MAP kinase assay

14-days-old seedlings were grown for five days on MS plates and than transferred to liquid MS. Triplicates of two seedlings each were treated with water, 100 nM elf18 or 100 nM flg22 for 0, 5 and 15 min before being pooled for harvest. Seedlings were ground to fine powder in liquid nitrogen and solubilised in better lacus buffer [50 mM Tris-HCl pH 7.5; 100 mM NaCl; 15 mM EGTA; 10 mM MgCl_2_; 1 mM NaF; 1 mM Na_2_MoO_4_.2H_2_O; 0.5 mM NaVO_3_; 30 mM β-glycerophosphate; 0.1% IGEPAL CA 630; 100 nM calyculin A (CST); 0.5 mM PMSF; 1% protease inhibitor cocktail (Sigma, P9599)]. The extracts were centrifuged at 16,000×g, the supernatant cleared by filtering through Miracloth and 4xLDS loading buffer (Invitrogen) added. 40 µg of total protein was separated by SDS-PAGE and blotted onto PVDF membrane (Biorad). Immunoblots were blocked in 5% (w/v) BSA (Sigma) in TBS-Tween (0.1%) for 1–2 H. The activated MAP kinases were detected using anti-p42/44 MAPK primary antibodies (1∶1000, Cell Signaling Technology) overnight, followed by anti-rabbit-HRP conjugated secondary antibodies (Sigma).

### Seedling growth inhibition

Fresh harvested seeds were surface sterilized, sown on MS media, stratified for 2 days at 4°C in the dark and put in the light. Five-day-old seedlings were transferred into liquid MS with or without the indicated amount of peptide and incubated for eight further days. Dry weight of six replicates per treatment was measured using a precision scale (Sartorius) and blotted relative to untreated control.

### ROS burst assay

Eight leaf discs (4 mm diameter) of at least four 3–4 week plants were sampled using a cork borer and floated over night on sterile water. The following day the water was replaced with a solution of 17 mg/ml (w/v) luminol (Sigma) and 10 mg/ml horseradish peroxidase (Sigma) containing 100 nM elf18 or 100 nM flg22. Luminescence was captured either using a Varioskan Flash (Thermo Scientific) multiplate reader or Photek camera (East Sussex, UK). The amount of relative light units might differ depending on the light capturing apparatus used.

### Transient expression in *N. benthamiana*

The whole coding sequence without the stop codon of *FLS2*, *EFR*, *BAK1* and *BAK1-5* was PCR amplified using the primers given in Table S2 and cloned into the *pENTR-D/TOPO* vector using the pENTR Directional TOPO cloning kit (Invitrogen). The resulting clones were verified by restriction analysis and sequence. The kinase dead variants FLS2*, EFR* and BAK1* were generated by point mutagenesis using the primers given in Table S2 and sequence verified. The coding sequences of *FLS2*, FLS2*, *EFR* and EFR* were transferred into *pEarleyGate103* [74] using the method described for Gateway vectors generating C-terminal GFP-His-tag fusion constructs under the 35S promoter. The coding sequence of *BAK1*, BAK1* and *BAK1-5* were transferred into *pGWB14* generating C-terminal HA-tag fusion constructs under the 35S promoter. The *CERK1p::CERK1-3xHA* construct was previously published [75]. The *EFRp::EFR-3xHA* construct, containing own promoter plus coding region, was described previously with the exception of using *epiGreenB5* as binary vector [36]. All resulting constructs were verified by restriction analysis and transformed into *A. tumefaciens* strain GV3101.

The constructs of *BAK1p*::*BAK1-eGFP* or *BAK1p::BAK1-5-eGFP*, containing own promoter plus coding regions, were PCR amplified using primers given in Table S2. The resulting constructs were cloned into pCR-Blunt-II-TOPO (Invitrogen) and verified by sequencing. The inserts were released by digesting with BsmBI and BamHI (NEB) and ligated into *epiGreenB(eGFP)* digested with EcoRI and BamHI (NEB). Resulting constructs were verified by restriction analysis transformed into *A. tumefaciens* strain AglI containing the *pSOUP* helper plasmid.

*A. tumefaciens* containing the indicated constructs were grown in L medium supplemented with the appropriate antibiotics overnight. Cultures were spun down and resuspended in 10 mM MgCl_2_ to a final O.D._600_ = 0.2-0.5. The indicated cultures were mixed 1∶1 and syringe infiltrated into 3-week-old *N. benthamiana* leaves. After 2 dpi whole leaves were vacuum infiltrated with water or 100 nM of the indicated peptide, incubated for 5 min and harvested by freezing in liquid nitrogen.

### Protein extraction and immunoprecipitation in *N. benthamiana*

Leaves were ground to fine powder in liquid nitrogen and 5 ml extraction buffer [50 mM Tris-HCl pH 7.5; 150 mM NaCl; 10% glycerol; 10 mM DTT; 10 mM EDTA; 1 mM NaF; 1 mM Na_2_MoO_4_.2H_2_O; 1% (w/v) PVPP; 1% (v/v) P9599 protease inhibitor cocktail (Sigma); 1% (v/v) IGEPAL CA-630 (Sigma)] added. Samples were cleared by centrifugation at 16.000×g for 15 min at 4°C and adjusted to 2 mg/ml total protein concentration. Immunoprecipitation were performed on 1.5 ml total protein by adding 20 µl GFPTrap-A beads (Chromotek) and incubation at 4°C for 3–4 H. Beads were washed 4 times with TBS containing 0.5% (v/v) IGEPAL CA-630, immunoprecipitates eluted with 30 µl 2xLDS (Invitrogen) and heating at 70°C for 10 min.

### SDS-PAGE and immunoblotting

SDS-gels were prepared with either 7.5 or 10% cross-linking. Gels were run at 80/150 V and proteins electroblotted onto PVDF membrane at 235 mA (Biorad). Membranes were rinsed in TBS and blocked in 5% (w/v) nonfat milk powder in TBST 0.1% (w/v) for 1 H. Primary antibodies were diluted in blocking solution to the following concentration and incubated overnight: anti-GFP (AMS Biotechnology) 1∶5000; anti-BAK1 1∶500; anti-HA-HRP (Santa Cruz) 1∶2000; anti-FLS2 1∶1000; anti-BRI1 1∶1000. Membranes were washed 3 times in TBST 0.1% (w/v) before 1 hour incubation with secondary antibodies anti-rabbit-HRP (Sigma) 1∶5000 or anti-rabbit-HRP (Ebioscience) 1∶5000. Signals were visualized using chemiluminescent substrate (Lumigen ECL, GE Healthcare) before exposure to film (AGFA CP-BU).

### Protein extraction and immunoprecipitation in Arabidopsis

Leaves were ground to fine powder in liquid nitrogen and extraction buffer [50 mM Tris-HCl pH 7.5; 150 mM NaCl; 10% glycerol; 5 mM DTT; 2 mM EDTA; 1 mM NaF; 1 mM Na_2_MoO_4_.2H_2_O; 1 mM PMSF (Sigma); 5 mM Na_3_VO_4_, 1% (v/v) P9599 protease inhibitor cocktail (Sigma); 1% (v/v) IGEPAL CA-630 (Sigma)] added. Samples were cleared by centrifugation at 16.000×g for 15 min at 4°C and adjusted to 2 mg/ml total protein concentration. Immunoprecipitations were performed on 1.5 ml total protein by adding 20 µl true-blot anti-rabbit Ig beads (Ebioscience), 15 µl antibody and incubation at 4°C for 3–4 H. Beads were washed 4 times with TBS containing 0.5% (v/v) IGEPAL CA-630, immunoprecipitates eluted with 50 µl 2xLDS (Invitrogen) and heated at 70°C for 10 min.

## Supporting Information

Figure S1 *bak1-5*, but not *bak1-4*, is strongly impaired in flg22- and elf18-induced SGI. SGI of Col-0, *bak1-4* and *bak1-5* in the presence of 1 µM flg22 or elf18. Fresh weight is represented relative to untreated control. Results are average ± s.e (n = 6). This experiment was repeated three times with similar results.
(PDF)

Figure S2 BAK1-5 is causative for the reduced elf18-induced ROS burst and behaves in a semi-dominant manner. A. Expression of BAK1 and BAK1-5 in transgenic plants in the *bak1-4* background. Immunoblot of total protein from Col-0, *bak1-4 pBAK1::BAK1*, *bak1-4 pBAK1::BAK1-5* and *bak1-4* using anti-BAK1 antibody. Immunoblot, upper panel; Coomassie colloidal blue stained membrane, lower panel. B. The *bak1-5* mutation is causative for the compromised elf18-induced ROS burst. ROS burst in leaves of Col-0, *bak1-4*, *bak1-5*, *bak1-4 pBAK1::BAK1* and *bak1-4 pBAK1::BAK1-5* plants treated with 100 nM elf18. Results are average ± s.e. (n = 8). C. *bak1-5* behaves in a semi-dominant negative manner. ROS burst in leaves of Col-0, *bak1-4*, *bak1-5*, *bak1-5×bak1-4* F1 and *bak1-5*×Col-0 F1 plants treated with 100 nM elf18. Results are average ± s.e. (n = 8). These experiments were repeated at least twice with similar results.
(PDF)

Figure S3 Reduced steady-state defence genes expression in *bak1-5*. Gene expression of *At2g17740* (left), *CYP81F2* (middle) and *At1g51890* (right) in seedlings of Col-0, *bak1-4* and *bak1-5* was measured by qPCR analysis. Results are average ± s.e. (n = 3). This experiment was repeated four times with similar results.
(PDF)

Figure S4 The expression of BAK1-5 compromises disease resistance to *Pto* DC3000 *COR^−^*. Five week old plants Col-0, *bak1-4*, *bak1-5*, *bak1-4 pBAK1::BAK1* and *bak1-4 pBAK1::BAK-5* were spray-infected with *Pto* DC3000 *COR^−^* O.D._600 nm_ = 0.2, covered at high humidity for 3 days and left for another 2 days for disease symptoms to develop. Scale bar represents 4 cm.
(PDF)

Figure S5 The expression of BAK1-5 rescues the semi-dwarf phenotype of *bak1-4*. Picture of representative individuals of five-week-old Col-0, *bak1-4*, *bak1-5*, *bak1-4 pBAK1::BAK1* and *bak1-4 pBAK1::BAK-5* plants grown under short-day conditions. Scale bar represents 4 cm. This experiment was repeated at least three times with similar results.
(PDF)

Figure S6 *bkk1-1 bak1-5* does not show any early senescence phenotypes. Picture of representative individuals of six-week-old Col-0, *bak1-4*, *bak1-5*, *bkk1-1* and *bak1-5 bkk1-1* plants grown under short-day conditions. Scale bar represents 5 cm. This experiment was repeated twice with similar results.
(PDF)

Figure S7 BAK1-5 shows an enhanced interaction with FLS2. Co-immunoprecipitation of BAK1 or BAK1-5 with FLS2 in Col-0 or *bak1-5* plants treated or not with 100 nM flg22 for 5 min, respectively. Total proteins (T) were subjected to immunoprecipitation (IP) with anti-BAK1 antibodies and IgG beads followed by immunoblot analysis using anti-FLS2 or anti-BAK1 antibodies. This experiment was repeated twice with similar results.
(PDF)

Figure S8 BAK1 or BAK1-5 does not interact with CERK1. Co-immunoprecipitation CERK1-HA_3_ with either BAK1-GFP or BAK1-5-GFP after transient expression in *N. benthamiana* leaves. Leaves were treated or not with 100 mg/mL chitin for 5 min. Total protein (T) was subjected to immunoprecipitation with GFP-Trap beads followed by immunoblot analysis using anti-GFP or anti-HA antibodies. The asterisk indicates an unspecific band.
(PDF)

Figure S9 BAK1-5 display an approximate three-fold reduction in kinase activity. Relative kinase activity measured as auto-phosphorlyation level of BAK1 or BAK1-5, respectively.
(PDF)

Figure S10 FLS2 is an inactive kinase *in vitro. In vitro* kinase assay using His or N-terminal His-tagged FLS2, FLS2*, BRI1 and BRI1* CD. Note that ten times more FLS2 and FLS2* CD was loaded compared to BRI1 and BRI1* CD. Autoradiogram, upper panel; Coomassie colloidal blue stained membrane, lower panel
(PDF)

Figure S11 Expression of BAK1, BAK1-5, BAK1*, and BAK1-5* in transgenic plants in the *bak1-4* background. Immunoblot of total proteins from Col-0, *bak1-4 pBAK1::BAK1*, bak1-4 pBAK1::BAK1*, *bak1-4 pBAK1::BAK1-5*, pBAK1::BAK1-5* and *bak1-4* using anti-BAK1 antibodies. Immunoblot, upper panel; Coomassie colloidal blue stained membrane, lower panel.
(PDF)

Figure S12 The kinase activity of EFR is required for elf18-induced ROS burst. ROS burst in *N. benthamiana* leaves transiently expressing *FLS2-GFP-His*, *EFR-GFP-His*, or EFR*-GFP-His treated with 100 nM elf18. Results are average ± s.e. (n = 8).
(PDF)

Table S1 The quantitative kinase out-put of BAK1 is not correlated with its ability to function in PTI or BR signaling pathways. The number of “−” indicates the severity of impairment of BAK1 specific function. ^a^ *in vitro* kinase activity of BAK1 variants, relative impairment partially approximated. ^b^ Impairment in PTI signaling was only measured as the respective BAK1 variant's ability to rescue the impairment of *bak1-4 bkk1-1* in flg22-triggered SGI. ^1^ ref. Wang et *al.* 2008 [21]. ^2^ ref. Oh et *al.* 2010 [34]. ^3^ ref. Li et *al.* 2002 [19], Wang et *al.* 2008 [21] and present study.
(DOC)

Table S2 Primers used in this study.
(DOC)
