# Supplementary figures and images for: Phosphorylation-Dependent Differential Regulation of Plant Growth, Cell Death, and Innate Immunity by the Regulatory Receptor-Like Kinase BAK1
Source: PLoS Genet. 2011 Apr 28;7(4):e1002046. doi: 10.1371/journal.pgen.1002046 (PMC3085482; doi:10.1371/journal.pgen.1002046)

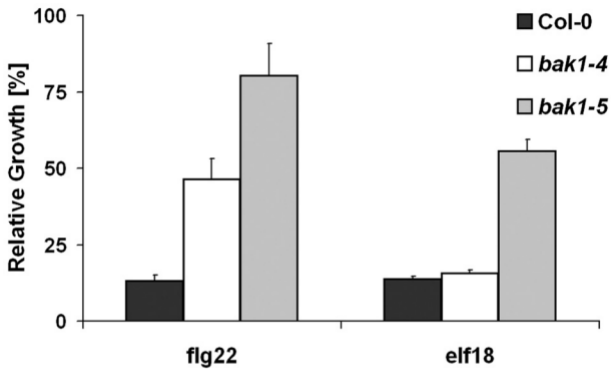

Supplement: Figure S1 — bak1-5, but not bak1-4, is strongly impaired in flg22- and elf18-induced SGI. SGI of Col-0, bak1-4 and bak1-5 in the presence of 1 µM flg22 or elf18. Fresh weight is represented relative to untreated control. Results are average ± s.e (n = 6). This experiment was repeated three times with similar results. (PDF) [file pgen.1002046.s001.pdf]

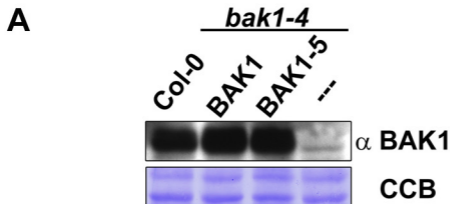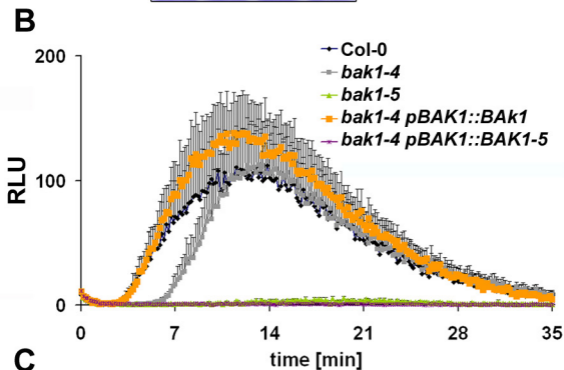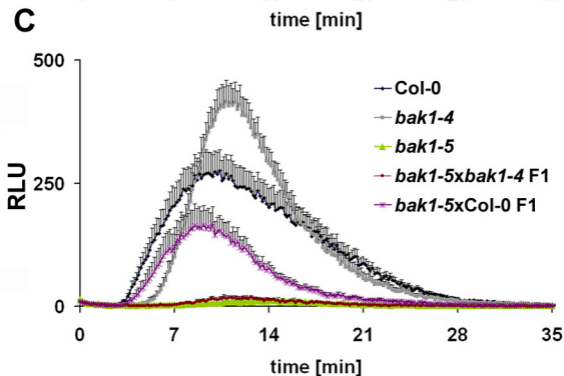

Supplement: Figure S2 — BAK1-5 is causative for the reduced elf18-induced ROS burst and behaves in a semi-dominant manner. A. Expression of BAK1 and BAK1-5 in transgenic plants in the bak1-4 background. Immunoblot of total protein from Col-0, bak1-4 pBAK1::BAK1, bak1-4 pBAK1::BAK1-5 and bak1-4 using anti-BAK1 antibody. Immunoblot, upper panel; Coomassie colloidal blue stained membrane, lower panel. B. The bak1-5 mutation is causative for the compromised elf18-induced ROS burst. ROS burst in leaves of Col-0, bak1-4, bak1-5, bak1-4 pBAK1::BAK1 and bak1-4 pBAK1::BAK1-5 plants treated with 100 nM elf18. Results are average ± s.e. (n = 8). C. bak1-5 behaves in a semi-dominant negative manner. ROS burst in leaves of Col-0, bak1-4, bak1-5, bak1-5×bak1-4 F1 and bak1-5×Col-0 F1 plants treated with 100 nM elf18. Results are average ± s.e. (n = 8). These experiments were repeated at least twice with similar results. (PDF) [file pgen.1002046.s002.pdf]

Relative expression

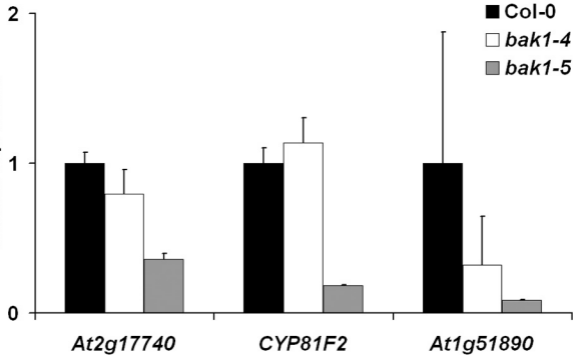

Supplement: Figure S3 — Reduced steady-state defence genes expression in bak1-5. Gene expression of At2g17740 (left), CYP81F2 (middle) and At1g51890 (right) in seedlings of Col-0, bak1-4 and bak1-5 was measured by qPCR analysis. Results are average ± s.e. (n = 3). This experiment was repeated four times with similar results. (PDF) [file pgen.1002046.s003.pdf]

**Col-0**

***bak1-4***

***bak1-5***

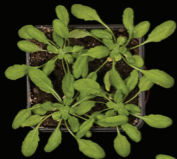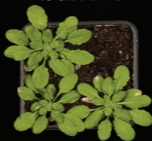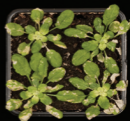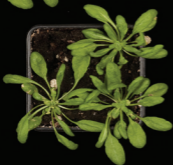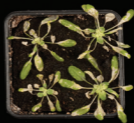

***BAK1***

***BAK1-5***

***bak1-4***

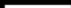

Supplement: Figure S4 — The expression of BAK1-5 compromises disease resistance to Pto DC3000 COR−. Five week old plants Col-0, bak1-4, bak1-5, bak1-4 pBAK1::BAK1 and bak1-4 pBAK1::BAK-5 were spray-infected with Pto DC3000 COR− O.D.600 nm = 0.2, covered at high humidity for 3 days and left for another 2 days for disease symptoms to develop. Scale bar represents 4 cm. (PDF) [file pgen.1002046.s004.pdf]

**Col-0**

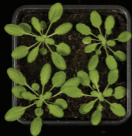

***bak1-4***

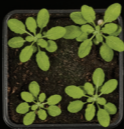

***bak1-5***

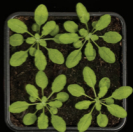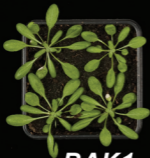

***BAK1***

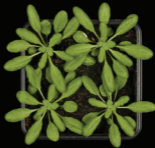

***BAK1-5***

***bak1-4***

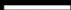

Supplement: Figure S5 — The expression of BAK1-5 rescues the semi-dwarf phenotype of bak1-4. Picture of representative individuals of five-week-old Col-0, bak1-4, bak1-5, bak1-4 pBAK1::BAK1 and bak1-4 pBAK1::BAK-5 plants grown under short-day conditions. Scale bar represents 4 cm. This experiment was repeated at least three times with similar results. (PDF) [file pgen.1002046.s005.pdf]

**Col-0**

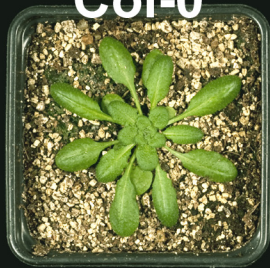

***bak1-4***

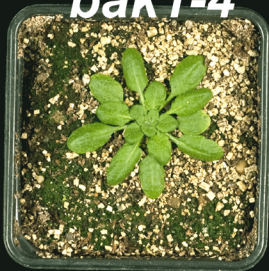

***bak1-5***

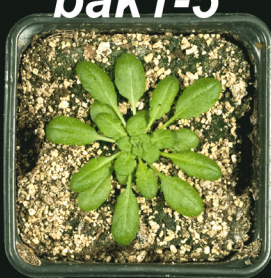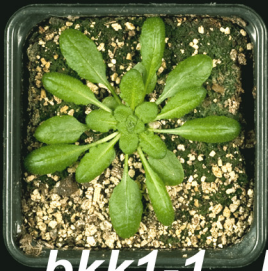

***bkk1-1***

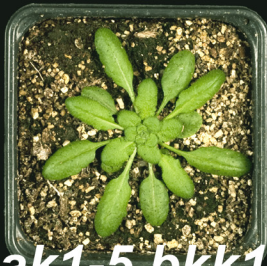

***bak1-5 bkk1-1***

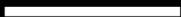

Supplement: Figure S6 — bkk1-1 bak1-5 does not show any early senescence phenotypes. Picture of representative individuals of six-week-old Col-0, bak1-4, bak1-5, bkk1-1 and bak1-5 bkk1-1 plants grown under short-day conditions. Scale bar represents 5 cm. This experiment was repeated twice with similar results. (PDF) [file pgen.1002046.s006.pdf]

IP:

$\alpha$ BAK1

-flg22

+flg22

BAK1 BAK1-5 BAK1 BAK1-5

IP

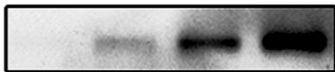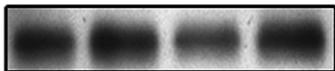

T

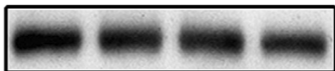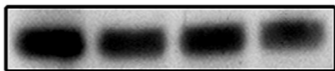

WB:

$\alpha$  FLS2

$\alpha$  BAK1

$\alpha$  FLS2

$\alpha$  BAK1

Supplement: Figure S7 — BAK1-5 shows an enhanced interaction with FLS2. Co-immunoprecipitation of BAK1 or BAK1-5 with FLS2 in Col-0 or bak1-5 plants treated or not with 100 nM flg22 for 5 min, respectively. Total proteins (T) were subjected to immunoprecipitation (IP) with anti-BAK1 antibodies and IgG beads followed by immunoblot analysis using anti-FLS2 or anti-BAK1 antibodies. This experiment was repeated twice with similar results. (PDF) [file pgen.1002046.s007.pdf]

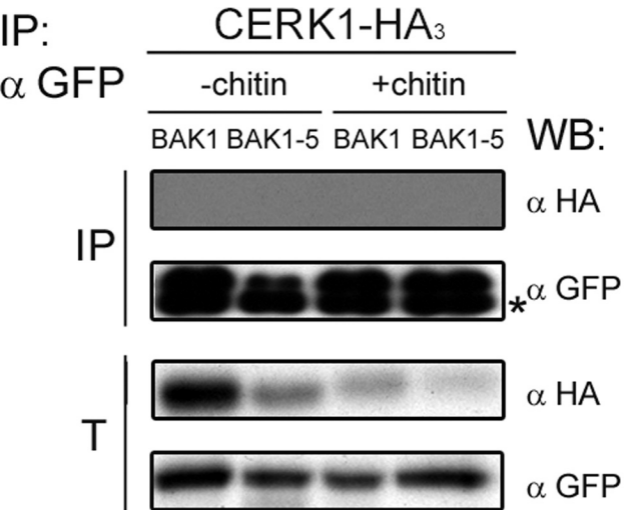

Supplement: Figure S8 — BAK1 or BAK1-5 does not interact with CERK1. Co-immunoprecipitation CERK1-HA3 with either BAK1-GFP or BAK1-5-GFP after transient expression in N. benthamiana leaves. Leaves were treated or not with 100 mg/mL chitin for 5 min. Total protein (T) was subjected to immunoprecipitation with GFP-Trap beads followed by immunoblot analysis using anti-GFP or anti-HA antibodies. The asterisk indicates an unspecific band. (PDF) [file pgen.1002046.s008.pdf]

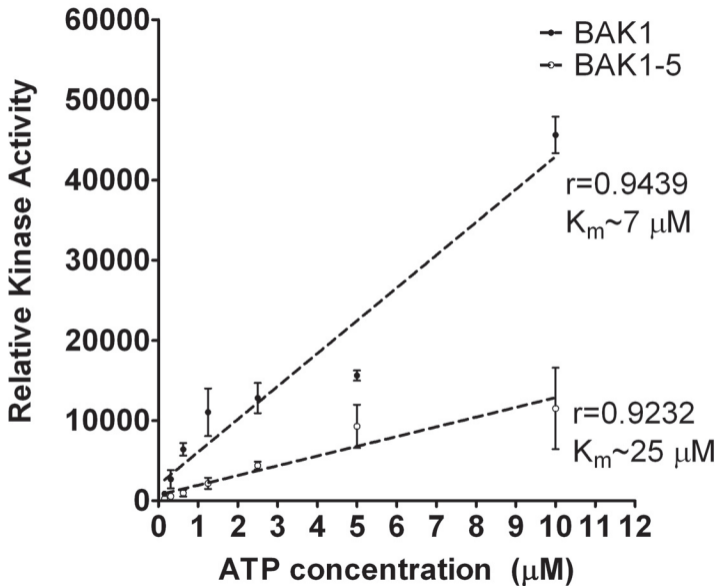

Supplement: Figure S9 — BAK1-5 display an approximate three-fold reduction in kinase activity. Relative kinase activity measured as auto-phosphorlyation level of BAK1 or BAK1-5, respectively. (PDF) [file pgen.1002046.s009.pdf]

His FLS2 FLS2\* BRI1 BRI1\*

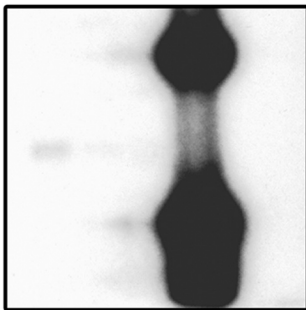

◀ BRI1

◀ FLS2

◀ MBP

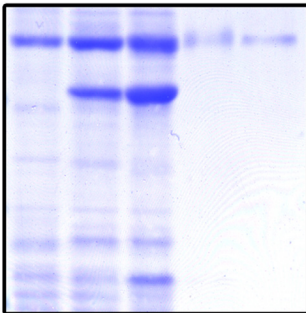

Supplement: Figure S10 — FLS2 is an inactive kinase in vitro. In vitro kinase assay using His or N-terminal His-tagged FLS2, FLS2*, BRI1 and BRI1* CD. Note that ten times more FLS2 and FLS2* CD was loaded compared to BRI1 and BRI1* CD. Autoradiogram, upper panel; Coomassie colloidal blue stained membrane, lower panel (PDF) [file pgen.1002046.s010.pdf]

*bak1-4*

Col-0  
BAK1  
BAK1-5  
BAK1\*  
BAK1-5\*  
---

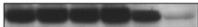

α BAK1

CCB

Supplement: Figure S11 — Expression of BAK1, BAK1-5, BAK1*, and BAK1-5* in transgenic plants in the bak1-4 background. Immunoblot of total proteins from Col-0, bak1-4 pBAK1::BAK1, bak1-4 pBAK1::BAK1*, bak1-4 pBAK1::BAK1-5, pBAK1::BAK1-5* and bak1-4 using anti-BAK1 antibodies. Immunoblot, upper panel; Coomassie colloidal blue stained membrane, lower panel. (PDF) [file pgen.1002046.s011.pdf]

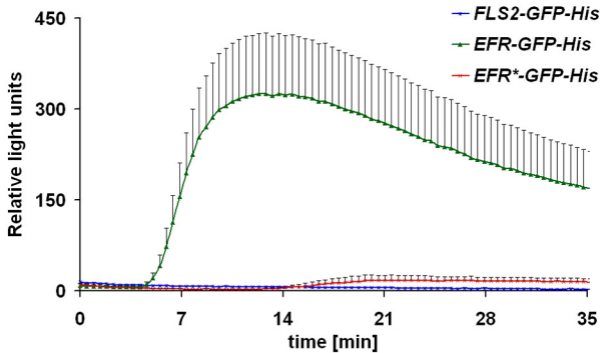

Supplement: Figure S12 — The kinase activity of EFR is required for elf18-induced ROS burst. ROS burst in N. benthamiana leaves transiently expressing FLS2-GFP-His, EFR-GFP-His, or EFR*-GFP-His treated with 100 nM elf18. Results are average ± s.e. (n = 8). (PDF) [file pgen.1002046.s012.pdf]
