# Supplementary material for: Phosphorylation-Dependent Differential Regulation of Plant Growth, Cell Death, and Innate Immunity by the Regulatory Receptor-Like Kinase BAK1
Source: PLoS Genet. 2011 Apr 28;7(4):e1002046. doi: 10.1371/journal.pgen.1002046 (PMC3085482; doi:10.1371/journal.pgen.1002046)
Supplement: Table S1 — The quantitative kinase out-put of BAK1 is not correlated with its ability to function in PTI or BR signaling pathways. The number of “−” indicates the severity of impairment of BAK1 specific function. a in vitro kinase activity of BAK1 variants, relative impairment partially approximated. b Impairment in PTI signaling was only measured as the respective BAK1 variant's ability to rescue the impairment of bak1-4 bkk1-1 in flg22-triggered SGI. 1 ref. Wang et al. 2008 [21]. 2 ref. Oh et al. 2010 [34]. 3 ref. Li et al. 2002 [19], Wang et al. 2008 [21] and present study. (DOC) [file pgen.1002046.s013.doc]

|  | **BAK1** | **BAK1-5**  **(C408Y)** | **BAK1**  **(Y610F)**^2^ | **BAK1**  **(T450A)**^1^ | **BAK1**  **(T449A)**^1^ | **BAK1***  **(D418N)**^3^ |
| --- | --- | --- | --- | --- | --- | --- |
| **PTI** | **WT** | **---** | **WT**^b^ | **-**^b^ | **WT**^b^ | **--** |
| **BR** | **WT** | **WT** | **--** | **-** | **WT** | **---** |
| **Cell-**  **death** | **WT** | **WT** | **WT** | **WT** | **WT** | **-** |
| **kinase**  **activity**^a^ | **WT** | **-** | **-/--** | **--** | **---** | **----** |

**Table S1: The quantitative kinase output of BAK1 is not correlated with its ability to function in PTI or BR signaling pathways.**

The number of “- “ indicates the severity of impairment. of BAK1 specific function.

^a^ *In vitro* kinase activity of BAK1 variants, relative impairment partially approximated.

^b^ Impairment in PTI signaling was only measured as the respective BAK1 variant ability to rescue the impairment of *bak1-4 bkk1-1* in flg22-triggered SGI.

^1^ ref. Wang et *al.* 2008.

^2^ ref. Oh et *al.* 2010.

^3^ ref. Li et *al.* 2002, Wang et *al.* 2008 and present study.
