# Supplementary material for: Phosphorylation-Dependent Differential Regulation of Plant Growth, Cell Death, and Innate Immunity by the Regulatory Receptor-Like Kinase BAK1
Source: PLoS Genet. 2011 Apr 28;7(4):e1002046. doi: 10.1371/journal.pgen.1002046 (PMC3085482; doi:10.1371/journal.pgen.1002046)
Supplement: Table S2 — Primers used in this study. (DOC) [file pgen.1002046.s014.doc]

| **Primer name** | **5' to 3' sequence** |
| --- | --- |
| BAK1_4_F | CATGACATCATCATCATTCGC |
| BAK1_4_R | ATTTTGCAGTTTTGCCAACAC |
| BKK1-1_F | TGGCTCAGAAGAAAACCACAG |
| BKK1-1_R | CTGCTCCACTTCTGTTTCCAC |
| BRI1-301_F | CATCGAAATCTTGTGCCTCTT |
| BRI1-301_R | GAACGTAACCCGGTGTACCA |
| BAK1_dCAPS_F | AAGAGGGCTTGCGTATTTACATGATCAGT |
| BAK1_dCAPS_R | GAGGCGAGCAAGATCAAAAG |
| EFR_KD_F | AAGTTCTGTTTCAGGGCCCGGCCAGTGATGGTAACCCATC |
| EFR_KD_R | ATGGTCTAGAAAGCTTTACATAGTATGCATGTCCGTATTTAACATC |
| FLS2_KD_F | AAGTTCTGTTTCAGGGCCCGGAAAATTCATCAGAGTCCTCATTACCG |
| FLS2_KD_R | ATGGTCTAGAAAGCTTTAAACTTCTCGATCCTCGTTACGATC |
| BRI1_KD_F | AAGTTCTGTTTCAGGGCCCGGGTAGAGAGATGAGGAAGAGACG |
| BRI1_KD_R | ATGGTCTAGAAAGCTTTATAATTTTCCTTCAGGAACTTCTTTTATAC |
| BAK1_gateway_start_F | CACCATGGAACGAAGATTAATGATCCCTTGCTTC |
| BAK1_nostop_R | TCTTGGACCCGAGGGGTATTCGTTTTC |
| EFR_gateway_start_F | CACCATGAAGCTGTCCTTTTCACTTG |
| EFR_nostop_R | CATAGTATGCATGTCCGTATTTAACATCC |
| BRI1_gateway_start_F | CACCATGAAGACTTTTTCAAGCTTCTTTCT |
| BRI1_nostop_R | TAATTTTCCTTCAGGAACTTCTTTTATAC |
| FLS2_gateway_start_F | CACCATGAAGTTACTCTCAAAGACCTTTTTG |
| FLS2_nostop_R | AACTTCTCGATCCTCGTTACGATC |
| BAK1_(C408Y)_F | GAGGGCTTGCGTATTTACATGATCATTACGACCCAAAGATTATTCATCGAGATGTG |
| BAK1_(C408Y)_R | CACATCTCGATGAATAATCTTTGGGTCGTAATGATCATGTAAATACGCAAGCCCTC |
| BAK1_(D416N)_F | CCCAAAGATTATTCATCGAAATGTGAAAGCTGCAAATATTTTGTTG |
| BAK1_(D416N)_R | CAACAAAATATTTGCAGCTTTCACATTTCGATGAATAATCTTTGGG |
| EFR_(D848N)_F | GACCCTGTAGCTCACTGTAATATTAAGCCAAGCAACA |
| EFR_(D848N)_R | TGTTGCTTGGCTTAATATTACAGTGAGCTACAGGGTC |
| FLS2_(D997N)_F | GGTTTTCCCATCGTTCATTGTAATCTGAAGCCAGCTAATATACTC |
| FLS2_(D997N)_R | GAGTATATTAGCTGGCTTCAGATTACAATGAACGATGGGAAAACC |
| BRI1_(D1009N)_F | GTCCGCATATCATCCACAGAAACATGAAATCCAGTAATGTGTTG |
| BRI1_(D1009N)_R | CAACACATTACTGGATTTCATGTTTCTGTGGATGATATGCGGAC |

Table S2: Primers used in this study
